# Engineered Poly (amino acid) Hydrogel Synchronizes Sequential Infection Elimination and Osteogenic Activation for Single-stage Reconstruction of Infected Bone Defects

**DOI:** 10.7150/thno.121585

**Published:** 2026-01-01

**Authors:** Yutao Cui, Yuhang Tian, Minghan Dou, Shaorong Li, Yi Fan, Gan Wang, Chuangang Peng, Yanbing Wang, Dankai Wu

**Affiliations:** Orthopedic Medical Center, The Second Hospital of Jilin University, Changchun, China.

**Keywords:** antibacterial, osteogenesis, hydrogel, infected bone defect, single-stage treatment

## Abstract

**Rationale:** The treatment of infected bone defects is always a serious challenge in orthopedics. Infection control in the first stage followed by bone reconstruction in the second stage is the main clinical treatment process. The design of dual-functional materials with sequential antibacterial and bone-promoting properties according to the content and temporal characteristics of these two clinical tasks is a promising solution.

**Methods:** In this study, the hydrogel composed of poly (ethylene glycol)-poly (L-alanine N-carboxyanhydride-co-L-phenylalanine N-carboxyanhydride) was constructed by ring-opening polymerization, and then its osteogenic and antibacterial properties were obtained by BMP-2 peptide grafting and vancomycin loading, respectively, and the engineered bifunctional hydrogel was constructed by combining these two functional hydrogels for sequential treatment of infected bone defects. A series of *in vitro* characterization experiments, biocompatibility analysis and evaluation of antibacterial and osteogenic properties were conducted. The specific therapeutic effect *in vivo* of constructed hydrogel was assessed using a model of infectious bone defect in the radius of rabbits.

**Results:** Our results confirmed that the engineered hydrogel had the physicochemical properties suitable for clinical scenarios and rapid and sustained antibiotic release performance in the early stage, which resulted in significant inhibition of both Gram-positive and Gram-negative bacteria *in vitro*. Moreover, the hydrogel had appropriate cytocompatibility and demonstrated the ability to promote osteogenic differentiation of stem cells. By establishing an infected bone defect of radius in rabbit model, we confirmed that the hydrogel could accelerate the repair of infected bone defect by simultaneously achieving satisfactory infection control and bone reconstruction.

**Conclusions:** This study provides a time-efficient single-stage strategy for the effective treatment of infected bone defects by constructing a dual-functional hydrogel with sequential antibacterial and osteogenic properties, which provides promising directions for future clinical treatment improvement.

## Introduction

Infected bone defect caused by open fractures, implant-related infections, etc. can often lead to limb necrosis, dysfunction and even amputation, which is a challenge in clinical treatment 1. In the infection microenvironment, pathogens and persistent inflammatory conditions can exert detrimental effects on bone repair through multiple mechanisms. Specifically, they directly inhibit the osteogenic process by promoting the apoptosis of osteoblasts and their internalization into osteoblasts. Indirectly, these factors can disrupt the extracellular matrix, promote osteoclastogenesis, and disturb the dynamic balance between osteoblasts and osteoclasts, ultimately impairing the bone regeneration 2. Therefore, infection control and bone regeneration are two major problems to be solved in the treatment of infected bone defects. The clinical treatment of infected bone defects is typically divided into two stages. In the first stage, complete surgical debridement, local antibiotic-loaded bone cement occupation, and systemic antibiotic administration are employed to eradicate pathogenic bacteria in the infected site as thoroughly as possible. In the second stage, the bone cement is removed and the bone reconstruction is conducted through masquelet technique, bone transport technique, etc. 3, 4. However, multiple operations increase the treatment cycle and the pain of patients. In the bone reconstruction stage, autologous bone transplantation has problems such as limited bone volume and donor site complications, and bone transport also has shortcomings such as nail tract infection 5-7. Therefore, it would be clinically important if the treatment of infected bone defects could be improved to address infection control and bone remodeling in a single-stage of treatment.

With the advancement of functional biomaterials in orthopedics, it is anticipated that bone repair can be achieved in a single operation by substituting the current antibiotic bone cement with biodegradable materials that possess both antibacterial and osteopromoting properties. Among these materials, hydrogels show unique advantages in clinical application scenarios for infected bone defects due to their strong plasticity to perfectly fill irregular defect sites, porous matrix network to enable the delivery of functional drugs to regulate the microenvironment, and biodegradation to avoid secondary surgical removal 8, 9. Depending on the therapeutic needs, these hydrogels can possess one or more of functionalities such as antibacterial ability, angiogenesis, etc. 10, 11. Recent studies have also confirmed that functional hydrogels play a role in promoting the repair of infected bone defects 12, 13. However, these multifunctional hydrogels are difficult to meet the different needs in different treatment stages, because infected bone defects require infection control in the first stage followed by bone reconstruction.

Notably, poly (amino acid) hydrogels are more conducive to design to achieve sequential regulatory functionality. Poly (amino acid) hydrogel is a network polymer structure composed of amino acid monomers through techniques such as ring-opening polymerization (ROP), which has good biocompatibility because its degradation does not produce acidic products 14, 15. Poly (amino acid) hydrogels with methoxypolyethylene glycols (mPEG) as initiator are commonly used in orthopedic biomaterials because of their good temperature sensitivity, which make them appear in solution state at low temperature for drug delivery, and gradually change into gel state with the increase of temperature 16, 17. More importantly, the mPEG terminal groups and amino or carboxyl terminal groups of mPEG-poly (amino acid) hydrogels can be functionalized by reaction with other materials or functional groups such as osteogenic peptides and materials 18-20. The drug loading strategy determines drug release kinetics. Drugs loaded by physical blending usually have a more rapid drug release due to free diffusion, whereas functional groups by covalent grafting exert a more delayed action by comparison 21, 22. Engineered hydrogels can be formed by combining mPEG-poly (amino acid) hydrogels with different loading modes, which can effectively play different roles at each stage and sequentially regulate the microenvironment of infected bone defects at the same time.

**Scheme 1:**
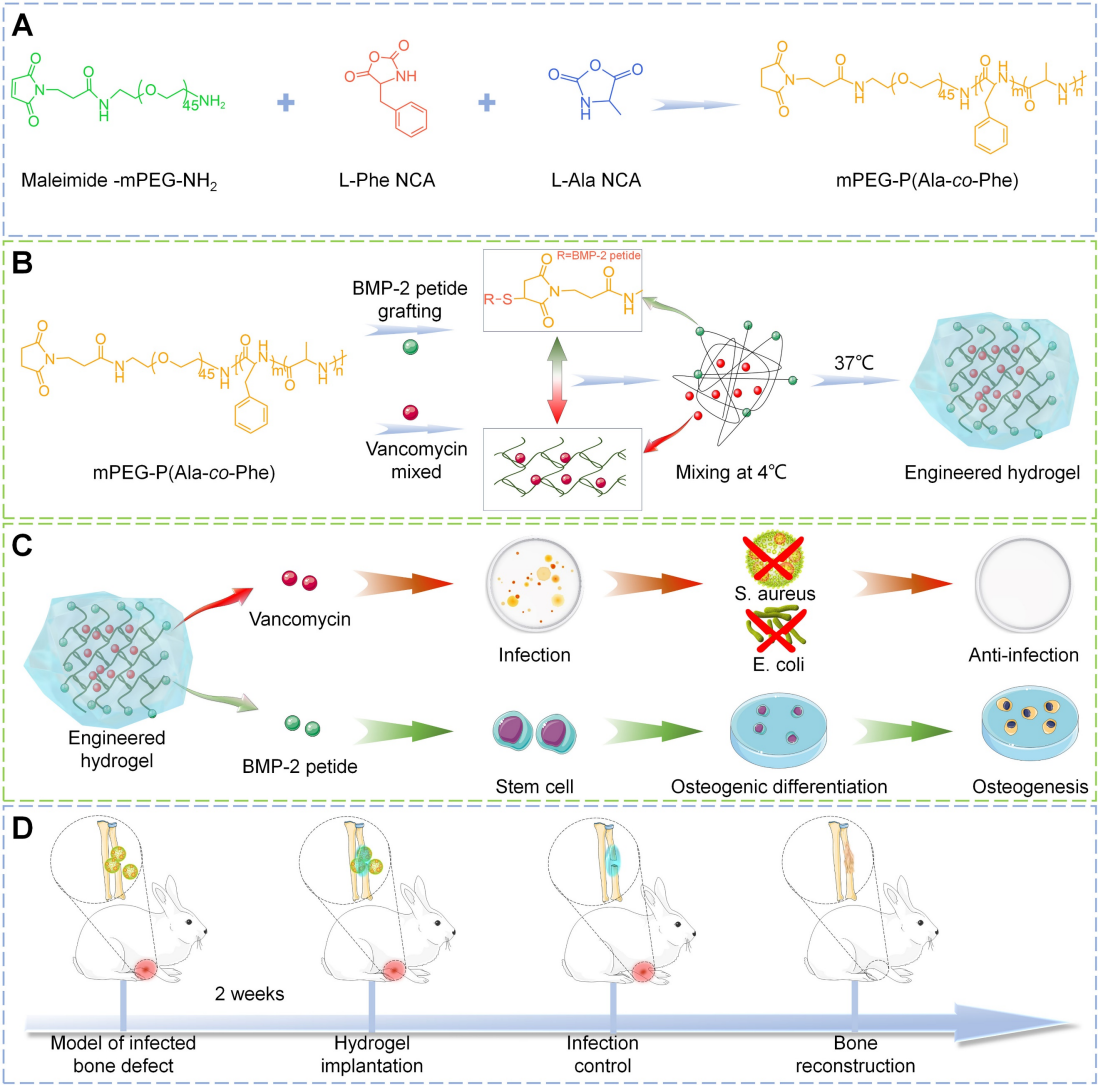
Demonstration of the construction of engineered hydrogels in this study (A-B) and its antibacterial and osteopromoting effects *in vitro* (C) and sequential infection control and bone re construction *in vivo* (D).

Herein, in this study, a multi-block polymer thermosensitive hydrogel was synthesized by ROP of L-alanine N-carboxyanhydride (Ala-NCA) and L-phenylalanine N-carboxyanhydride (Phe-NCA) with amino group of maleimide mPEG as initiator. Then, vancomycin, one of the most commonly used antibiotics in clinical practice 23, is encapsulated into the hydrogel matrix to create a rapid-response antibacterial system for early drug release. The osteogenic peptides derived from the knuckle epitope functional region of bone morphogenetic protein-2 (BMP-2) 24, the most commonly used growth factor in clinical practice 25, 26, were grafted onto the hydrogel by Michael addition reaction to construct a relatively delayed osteogenic promoting system. Engineered hydrogels were synthesized by the combination of antibacterial and osteogenic systems, which exhibit suitable structural stability, thermosensitivity, drug release profile, and biocompatibility. *In vitro* experiments showed that the engineered hydrogel could kill both Gram-positive and Gram-negative bacteria and significantly promote the osteogenic differentiation of bone marrow mesenchymal stem cells (BMSC). To further investigate the efficacy *in vivo*, the engineered hydrogel was implanted into the rabbit model of infected radial bone defect. As a functional spacer, the engineered hydrogel controlled the local infection state and improved the osteogenic microenvironment to promote bone repair (**Scheme 1**). Therefore, this work provides a promising single-stage approach to infected bone defect treatment, which realize bone repair by sequentially efficient sterilization and modulation of osteogenic differentiation.

## Materials and Methods

### Materials

The BMP-2-derived peptide (sequence: CKIPKASSVPTELSAISTLYL) was synthesized by GL Biochemistry (Shanghai, China). The maleimide-mPEG, triphosgene, L-Ala and L-Phe were purchased from Aladdin Scientific (Shanghai, China). Low glucose Dulbecco's modified Eagle's medium (LG-DMEM) and fetal bovine serum (FBS) were purchased from Gibco (Grand Island, NY, USA). Penicillin streptomycin double antibody and trypsin-EDTA (0.05% trypsin and 0.02% EDTA) solution were bought from Sigma-Aldrich (Shanghai, China). Cell Counting kit-8 (CCK-8), calcein-AM-PI staining kit, RIPA lysis buffer, BCIP/NBT Alkaline phosphatase (ALP) Colour Development Kit and ALP assay kit were obtained from Beyotime Co., Ltd. (Shanghai, China). Phosphate-buffered saline (PBS), DAPI solution, TRITC Phalloidin and 4% paraformaldehyde were purchased from Solarbio (Beijing, China). Osteogenic medium of rabbit BMSCs and alizarin red staining were obtained from Cyagen (Santa Clara, USA). A SPARKeasy Cell RNA Kit was purchased from Sparkjade Biotechnology Co., Ltd. (Shandong, China). ABScript Ш RT Master Mix for qPCR with gDNA Remover and Universal SYBR Green Fast qPCR Mix were purchased from Abclonal Co., Ltd. (Wuhan, China).

### Preparation of the engineered hydrogel

Using anhydrous tetrahydrofuran as solvent, L-Ala and L-Phe were respectively reacted with triphosgene at 60 ℃ for 1 h in anhydrous environment, and then the products were purified to obtain L-Ala-NCA and L-Phe-NCA. The copolymers were synthesized by ROP of L-Ala NCA and L-Phe NCA using maleamide-mPEG-NH_2_ as macromolecular initiator (**Scheme 1**). Briefly, L-Ala NCA, L-Phe NCA were dissolved in DMF, followed by the addition of maleamides -mPEG to the solution, and after 3 days of reaction at room temperature, a 5-fold volume of diethyl ether was poured into the resulting solution to obtain copolymer precipitates. The final product mPEG-poly (Ala-co-Phe) hydrogel (PH) was subsequently obtained by lyophilization. For the preparation of hydrogel, the lyophilized PH powder was dissolved in sterilized PBS (6 wt%) at 4 ℃ followed by constantly stirring until the mixture was homogeneous and free of significant insoluble. The temperature of the solution was then raised to 37 ℃ to obtain the hydrogel.

Vancomycin was mixed into the hydrogel solution (3wt%) at 4 ℃ and heated to 37 ℃ to obtain vancomycin-loaded hydrogel (P-VH). The BMP-2 grafted hydrogels were fabricated by the Michael addition reaction between the sulfhydryl group of cysteine in the BMP-2 peptide and the maleamide-PEG in the hydrogel (P-BH). The engineered hydrogel (P-B-VH) in this study was obtained by mixing P-VH and P-BH at a 3:1 ratio.

### Characterization of the engineered hydrogel

#### Evaluation of chemical composition

In order to verify the structural accuracy, the synthesized L-Ala-NCA and L-Phe-NCA were analyzed by hydrogen nuclear magnetic resonance spectroscopy (^1^H NMR) using a 300 MHz nuclear magnetic resonance spectrometer (Bruker AVANCE III 600M). In addition, mass spectrometry analysis was performed for further identification.

To verify the accuracy of P-B-VH hydrogel synthesis, the mixture of PH and BMP-2 peptides before synthesis, the P-BH hydrogel after synthesis and the P-B-VH after synthesis were characterized by Fourier transformed infrared spectroscopy (FTIR). Briefly, after freeze-drying, the hydrogel was mixed with potassium bromide, and the infrared absorption spectrum was detected in an infrared spectrometer (Thermo Fisher Nicolet 6700) at a wavelength of 4000 to 600 cm^-1^ with a resolution of 4 cm^-1^.

#### Morphology analysis

The surface morphology of the PH and P-B-VH was measured via scanning electron microscope (SEM). The prepared hydrogel was frozen at -80 ℃ and then lyophilized at -70 ℃ for 48 h to ensure adequate drainage of the water from the hydrogels. Then the samples were gold sputtered coated and the SEM images of the hydrogel were captured. The pore characteristics of the hydrogel were subsequently evaluated using ImageJ software based on SEM images.

#### Mechanical characterization

The rheological properties of the hydrogels were determined using a rheometer (NETZSCH KINEXUS LAB) with a 25 mm diameter plate geometry and a 0.4 mm gap. In the temperature detection mode, the hydrogel storage modulus (G') and loss modulus (G") were measured in the range of 0-60 ℃. In the strain detection mode, the temperature was set at 37 ℃, and the hydrogel storage modulus (G') and loss modulus (G") were measured in the range of 0.1% to 1000% strain.

The compression modulus of the hydrogel was evaluated. The prepared hydrogel was placed on a platform of a mechanical testing system (Autograph CSS 44100 Universal Testing Machine, Changchun Material Testing Machine Research Institute, Changchun, China) and a vertical compressive force was applied to the samples at a rate of 5 mm/min. The stress and the deform of hydrogel were recorded.

Uniaxial tensile experiments were subsequently performed. The hydrogel was fixed on the upper and lower ends of the universal testing machine, and then uniaxial stretching was performed at a tensile rate of 2 mm/min. The tensile force and displacement were recorded.

The adhesive properties of the hydrogel to bone tissue were evaluated by affixing the hydrogel to the central region of two rabbit radial bones. The samples were mounted on the platform of the universal mechanical testing machine and subjected to shear testing at a constant rate of 10 mm/min to determine the adhesive strength and corresponding displacement.

#### Biodegradability and histocompatibility

To evaluate the degradation behavior, the hydrogel was subcutaneously injected into the dorsal skin of Sprague-Dawley rats. At predetermined time points, the residual hydrogels were recorded and the weight were measured. Moreover, skin tissues and organs including heart, kidney, liver, and spleen were obtained for H&E staining to observe the inflammation and toxicity of local and systemic tissues to evaluate the histocompatibility of the hydrogel.

#### *In vitro* vancomycin and BMP-2 peptide release profile

First, 1 mL of each group hydrogel was injected in the centrifuge tube with 4 mL of PBS solution (pH = 7.4). Then the tubes were placed in an incubator shaker with 37 ℃ and 100 rpm. At the appropriate time points, 200 μL of the released solution was removed and placed with an equivalent volume of fresh PBS. The concentration of vancomycin was determined by spectrophotometry. The absorbance of samples was analyzed at 280 nm. The concentration of BMP-2 peptide was quantified by the BCA method. Drug release rates at each time point were calculated.

### Cell viability, proliferation and morphology

The BMSCs were obtained from 1-week-old New Zealand white rabbits according to the previously study 27. The cells were suspended and cultured in LG-DMEM with 10% FBS and 1% penicillin and streptomycin under 5% CO_2_ and 37 ℃. The passage at three were prepared for use. The stem cell-related antigens of the extracted BMSC were identified by immunofluorescence. After fixing and blocking the cells, anti-CD34 and anti-CD90 antibodies derived from rabbits were added respectively. After incubating on ice for 30 min, ABflo® 488 and Alexa Fluor® 568-conjugated goat anti-rabbit IgG secondary antibody were added respectively. After nuclear staining by DAPI, the staining results were observed under a fluorescence microscope.

To determine the biocompatibility of engineered hydrogels, Calcein-AM/PI staining was performed. The BMSCs were co-cultured at 8×10^3^ cells/mL in 48-well plates with PH, P-VH, P-BH and P-B-VH hydrogels, the well without any hydrogel treatment was set as control group (CON). The Calcein -AM/PI staining solutions were prepared according to the manufacturer' s instructions to immerse the samples after seeding for 1 day and 3 days. After staining under dark conditions at room temperature for 30 min, the stained cells were observed under fluorescence microscope.

CCK-8 assay was performed to evaluate the effect of the engineered hydrogels on cell proliferation. After 1, 3, and 7 days of co-culture of the cells with the hydrogel in each group, 10% of the medium volume of CCK-8 reagent was added, and after 2 h of incubation, the absorbance of the cells in each group was measured at 450 nm using a Bio-Rad microplate reader.

To further study the effect on cell morphology, after 3 days of co-culture, the samples were washed twice with PBS and fixed with 4% paraformaldehyde for 30min and then permeabilized with 0.1% Triton X-100 for 5 min. Rhodamine-phalloidin and DAPI were used to stain F-actin and the nucleus, respectively. The staining image were observed by fluorescence microscope. And the number of myofilaments was quantitatively analyzed by ImageJ software.

### *In vitro* antibacterial activity tests

The antibacterial activity of the engineered hydrogel was assessed using Gram-negative *Escherichia coli* (*E. coli*, ATCC25922) and Gram-positive *Staphylococcus aureus* (*S. aureus*, ATCC25923). The inhibition zone test was used to assess the antibacterial properties. In brief, the PH, P-VH, P-BH and P-B-VH hydrogel were placed on agar plate with 1×10^7^ CFU mL^-1^ of *E. coli* and *S. aureus* respectively, then incubated for 24 h at 37 ℃ to calculate the zone of inhibition (ZOI).

Bacterial proliferation curve and plate count assay were performed to further evaluate the antimicrobial activity of hydrogel. The group only containing bacterial suspension with medium was used as the control (CON). The *E. coli* and *S. aureus* were incubated in the Luria-Bertani (LB) broth shaking at 37 ℃ and 120 rpm overnight. Then the suspension (1×10^7^ CFU mL^-1^) of the bacteria were co-cultured with CON, PH, P-VH, P-BH and P-B-VH for 96 h. At each 12 h, the 100 μL bacteria suspension was extracted into a 96-well culture plate and detected the optical density (OD) values at 600 nm. The corresponding bacterial growth curve was made. At the 24 h time point, the 100 μL suspension from each group was diluted (1×10^2^ CFU mL^-1^) and spread on LB agar plates and the number of the colonies on the plate was counted.

The effect of the P-B-VH hydrogel on biofilm formation was subsequently evaluated. A volume of 200 μL of *S. aureus* suspension at a concentration of 1 × 10⁹ CFU/mL was inoculated into 24-well plates. Following a 48-hour co-culture period with each respective material group, the samples were rinsed twice with PBS. The biofilms were then fixed with methanol and stained with 0.1% crystal violet for microscopic examination. Semi-quantitative assessment of biofilm formation was conducted by measuring the absorbance at 595 nm after eluting the crystal violet with 33% acetic acid.

### Effects on osteogenic differentiation *in vitro*

#### Transwell experiment

The Transwell experiment was first conducted to evaluate the recruitment effect of hydrogels on BMSC. Transwell chambers were placed into 24-well plates, with the upper chamber designated for BMSC culture and the lower chamber containing materials from the CON, PH, P-VH, P-BH, and P-B-VH groups, respectively. Following a 24 h incubation period, the upper chambers were retrieved, non-migrated cells were removed via PBS washing, and the migrated cells were fixed using 4% paraformaldehyde. Following staining with crystal violet, BMSC migration in each group was examined under a microscope. The number of migrated cells per square millimeter was semi-quantitatively analyzed using ImageJ software.

### ALP staining and activity evaluation

For the alkaline phosphatase (ALP) activity measurement and staining, BMSCs were seeded at a density of 8×10^4^ /well in a 6-well plate in the osteogenic medium (LG-DMEM medium supplemented with 50 μg/mL L-ascorbic acid, 10 mM β-glycerol phosphate and 10^-8^ M dexamethasone). After culturing for 7 days, the cells were fully lysed in RIPA lysis buffer and centrifuged. The supernatant was then added into a 96-well plate, and chromogenic substrate and diethanolamine buffer were sequentially added into each group of samples. After the mixed solution was incubated for 30 min at 37 ℃, the reaction stop solution was added. The absorbance of each group was measured at 405 nm to calculate the corresponding ALP activity. And for the ALP staining, the BMSCs were fixed with 4% paraformaldehyde and stained in the dark with the BCIP/NBT staining working solution (Beyotime Biotechnology) at room temperature. After incubation for 15 min, the stained samples were observed.

### ARS experiment

Alizarin red staining (ARS) was performed after the engineered hydrogel co-culturing with the BMSCs for 14 days. Cells were rinsed twice with PBS and then fixed with 4% paraformaldehyde for 30 min, ARS solution was added to each group of samples and stained for 30 min at room temperature. The excess staining solution was removed by PBS and the number of calcium nodules was observed. For semi-quantitative analysis, calcium nodules were dissolved with 10% cetylpyridinium chloride and the absorbance of the sample at 540 nm was measured.

### RT-qPCR

After co-culturing with hydrogels in each group for 7 days, the expression levels of osteogenic genes of runt-related transcription factor 2 (*Runx2*), *ALP*, osteopontin (*OPN*) and osteocalcin (*OCN*) were evaluated by real-time quantitative polymerase chain reaction (RT-qPCR). The total RNA were extracted by using a SPARKeasy Cell RNA Kit. The ABScript Ш RT Master Mix for qPCR with gDNA Remover was used to synthesized cDNA according to the manufacturer's instruction. Then, qPCR amplification and detection were performed on a LightCycler 480 using Universal SYBR Green Fast qPCR Mix. The primers of specific gene are shown in **Table S1**. The results were evaluated using the 2^-ΔΔCt^ method by normalizing to the expression levels of *GAPDH* as the housekeeping gene.

### *In vitro* immunofluorescence

*In vitro* immunofluorescence staining of Smad 1/5/8, phosphorylated Smad 1/5/8 (p-Smad 1/5/8) and Runx2 proteins was conducted to evaluate the molecular mechanism of the effect of P-B-VH hydrogel on osteogenic differentiation of BMSCs. After co-culturing BMSCs with each group in osteogenic induction medium for 3 days, the cells were fixed, permeated and blocked, and then incubated with primary anti-Smad 1/5/8, anti-p-Smad 1/5/8 and anti-Runx2 antibodies overnight at 4 °C. Subsequently, ABflo® 488-conjugated, Alexa Fluor® 568 conjugated and Cy3-conjugated goat anti-rabbit IgG secondary antibody was added and incubated in the dark at 37 °C for 1 h to label anti-Smad 1/5/8, anti-p-Smad 1/5/8 and anti-Runx2 primary antibodies respectively. The staining results were observed under a fluorescence microscope after the cell nuclei were stained with DAPI staining solution for 2 minutes.

### Western Blot

After co-culturing BMSCs in osteogenic induction medium for 3 days across all experimental groups, cells were lysed using RIPA lysis buffer supplemented with PMSF, protease inhibitor, and phosphatase inhibitor to extract total protein. The protein concentration was quantified using a BCA protein assay kit. Equal amounts of total protein were separated by SDS-PAGE and subsequently transferred onto PVDF membranes via electroblotting. Membranes were incubated overnight at 4 °C with primary antibodies against p-Smad1/5/8, Smad1/5/8, Runx2, and GAPDH at appropriate dilutions. Following incubation with secondary antibodies, immunoreactive bands were visualized using the JY-Clear ECL chemiluminescence gel imaging system.

### *In vivo* animal experiment

#### Establishment of infected bone defect model in rabbits

All animal studies were conducted in compliance with the National Institutes of Health's Guide for the Care and Use of Laboratory Animals (NIH Publication No. 85-23 Rev. 1985) and approved by the Animal Care and Use Ethics Committee of Jilin University. Forty rabbits were anesthetized with 3% pentobarbital sodium (30 mg/kg) by intraperitoneal injection. After the left forearm radius was exposed, a segment bone defect with a length of 1.5 cm was created in the middle of the radial shaft. Then the absorbable gelatin sponge soaked in the *S. aureus* suspension (10^7^ CFU/mL) was implanted into the defect site. No antibiotic treatment was performed postoperatively. At the predetermined intervals, venous blood was obtained through the auricular vein to test the levels of white blood cells (WBC) and neutrophile granulocyte (NE) in each group. After surgery for 2 weeks, the establishment of infected bone defect model was further verified by local signs of infection such as sinus formation, imaging evaluation, Giemsa staining and HE staining.

After confirming the successful construction of the infected bone defect model, rabbits were anesthetized and the defect area was debrided and irrigated, followed by five different treatments including control group without treatment (CON), implantation of PH, P-BH, P-VH and P-B-VH respectively at the defect area. The wound site was closed by suturing layer by layer and the rabbits were allowed free activity in the cage postoperatively.

### Bacterial culture at the site of infection

At week 4 and 8 after surgery, radius samples were collected from each group. The samples were immediately vibrated 5 min in PBS. Then, 100 μL of the soaking solution was transferred to the agar plate and evenly spread, then placed at 37 ℃ for 24 h to quantify the number of bacteria.

### Immunofluorescence staining

The inflammatory status of the defect site was assessed in each group. The radius samples collected at week 4 were fixed, decalcified, dehydrated, embedded and sectioned, and then incubated overnight with the rabbit-origin tumor necrosis factor α (TNF-α) and Interleukin-6 (IL-6) primary antibody at 4 °C. Then, FITC-labeled goat anti-rabbit IgG antibody and Cy3-labeled goat anti-rabbit IgG antibody were added and incubated at 37 ℃ in the dark for 1h to label the TNF-α and IL-6 primary antibody respectively. The staining results were observed under a fluorescence microscope after DAPI staining of the nuclei.

### Imaging assessment

Radiographs were taken every 4 weeks after surgery to assess the local bone regeneration. At week 8, samples were collected from each group for micro-computed tomography (micro-CT) (SkyScan 1076 scanner, Kontich, Belgium) scanning. The specimen was scanned at 40 kV, 250 μA current, and 18 μm image pixel size. The defect site was set as the region of interest (ROI), and the bone volume/tissue volume ratio (BV/TV), trabecular number (Tb. N), and trabecular separation (Tb. Sp) within the ROI was analyzed by CT Analyser 1.17.7.2 software. A 3D image of each sample was then reconstructed using the multimodal 3D visualization software (NRecon 1.7.1.0 software, Kontich, Belgium).

### Histological evaluation

The radial specimens collected were adequately fixed in a 4% paraformaldehyde solution. The specimens were subsequently decalcified, dehydrated with gradient ethanol, embedded in paraffin, and sectioned into 5 μm thick tissue sections. Hematoxylin and eosin (H&E) and Masson's trichrome staining were performed on the sample sections to evaluate the bone regeneration of the samples in each group, and Giemsa staining was performed to further evaluate the bacterial residues in each group. Stained samples were all observed using a digital microscope (DSX 500; Olympus Corporation, Tokyo, Japan).

### Immunohistochemistry

The expression of osteogenic proteins Runx2, ALP, OPN and OCN at the defect site was evaluated by immunohistochemistry. The radial sections were incubated overnight with anti-Runx2 primary antibody (ABclonal, Wuhan, China) and anti-OCN primary antibody (ABclonal, Wuhan, China) at 4 °C. After that, the sections were incubated with HRP-conjugated secondary antibodies at room temperature for 30 min. Thereafter, DAB chromogenic solution was applied, and the staining results were examined under a light microscope.

### Statistical analysis

All data are expressed as the mean ± standard deviation. One-way analysis of variance (ANOVA) followed by Bonferroni method was used to determine whether there were significant differences between two groups. A difference of p < 0.05 was considered statistically significant.

## Results and Discussions

### Characterization of the P-B-VH hydrogel

As shown in **Figure S1A**, the chemical shift peaks of L-Ala-NCA at about 6.34 ppm(a), 4.43 ppm(b) and 1.59 ppm(c) correspond to the hydrogen attached to the nitrogen atom (a), the hydrogen on the carbon attached to the amino group (b) and the hydrogen on the methyl group (c) in the molecular formula, respectively. Similarly, the chemical shift peaks of L-Phe-NCA at about 5.89 ppm (a), 4.56 ppm (b) and 7.33 ppm (e) correspond to the hydrogen attached to the nitrogen atom (a), the hydrogen on the carbon attached to the amino group (b) and the hydrogen on the benzene ring (c), respectively. Furthermore, the results of mass spectrometry (**Figure S1B-C**) also confirmed that the molecular masses of L-Ala-NCA and L-Phe-NCA were consistent with the predicted molecular masses. These results confirmed the successful synthesis of L-Ala-NCA and L-Phe-NCA.

**Figure 1:**
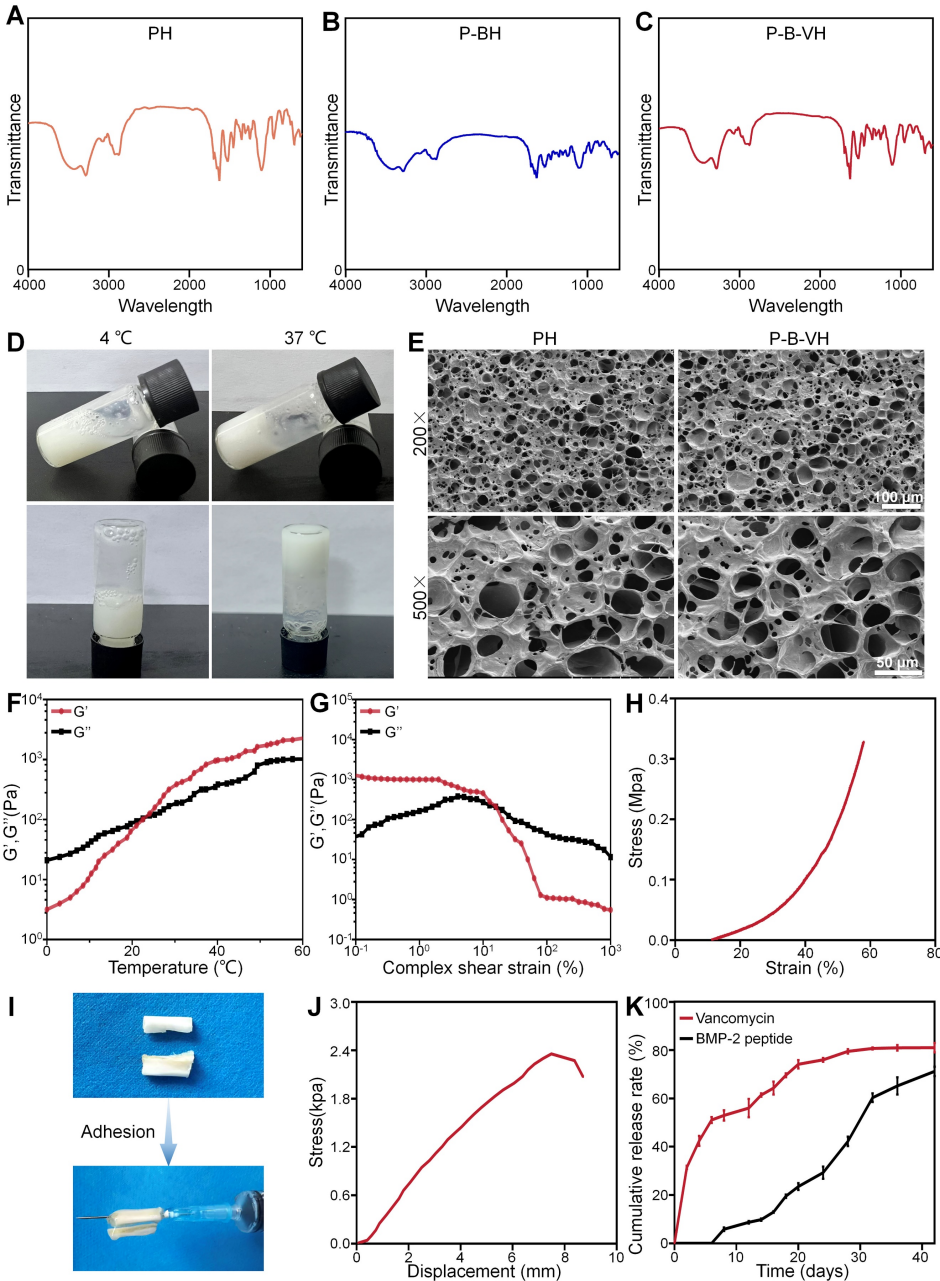
Construction and characterization of functionalized hydrogels: A-C. FT-IR analysis of PH, P-BH, and P-B-VH hydrogels; D. Schematic diagram of temperature-sensitive gelation of P-B-VH hydrogel; E. SEM images of PH and P-B-VH; F. Rheological detection of P-B-VH hydrogel over temperature; G. Rheological detection of P-B-VH hydrogel under varying strain; H. Evaluation of compressive strength of P-B-VH hydrogel; I-J. External image (I) and strength (J) of the adhesion ability of P-B-VH hydrogel. K. Drug release curve of P-B-VH hydrogel.

The chemical structure of P-B-VH hydrogel were characterized. As shown in **Figure 1A-C**, the FTIR of the mixture of PH hydrogel and BMP-2 peptide have the absorption of the thiol group in BMP-2 peptide at 2500 cm^-1^, and the absorption peak at 1104 cm^-1^ is attributed to the C-O-C bond in mPEG. The absorption peaks at 1626 cm^-1^ and 1524 cm^-1^ were attributed to the amide bond of the amino acid polymer. In addition, the stretching vibrations of the C=O and C=C bonds in the maleamide group also contribute to the absorption peak at 1626 cm^-1^. In contrast, the absorption of sulfhydryl group at 2500 cm^-1^ disappeared in P-BH and P-B-VH, and the absorption peak at 1626 cm^-1^ in P-BH decreased due to the Michael addition reaction of the C=C bond. The absorption peak of P-B-VH at 1626 cm^-1^ was similar to that of the mixture of PH and BMP-2 peptides due to the addition of P-VH hydrogel. These results confirmed the successful synthesis of P-B-VH hydrogel.

The P-B-VH hydrogel showed good thermosensitive gelation performance (**Figure 1D**). The morphological structure of the PH and P-B-VH hydrogels was evaluated by SEM, and the results showed that both PH and P-B-VH hydrogels exhibited similar cross-linked porous network structures (**Figure 1E**). **Figure S2A** presented the average pore sizes of the PH and P-B-VH groups were 103.8±20.7 μm and 100.1±23.1 μm, respectively. Both groups exhibited a relatively uniform pore size distribution. Among 30 randomly selected pore samples, pores smaller than 90 μm accounted for 16.7±5.5% in the PH group and 20.0±3.3% in the P-B-VH group. Pores ranging from 90 μm to 120 μm constituted 68.9±8.4% in the PH group and 63.3±5.8% in the P-B-VH group, respectively. Pores larger than 120 μm made up 14.4±3.8% in the PH group and 16.7±6.7% P-B-VH group, respectively. This temperature-sensitive property ensures the uniform distribution of vancomycin in the hydrogel matrix, and the interconnected pore structure in the hydrogel matrix facilitates the diffusion and release of the drug loaded in the matrix, and provides favorable conditions for the biodegradation of the hydrogel on the other hand 24, 28. Furthermore, previous studies have indicated that an interpore size range of 50-300 μm in the tissue engineering materials, which is comparable to that of human cancellous bone, will facilitate the growth of osteoblasts 27.

Rheological analysis was performed to further explore the structural stability of the P-B-VH hydrogel. As shown in **Figure 1F**, at low temperatures, the loss modulus G" was greater than the storage modulus G', and the hydrogel was in liquid phase. With the increase of temperature, G' rose rapidly. When the temperature exceeds 24.1 ℃, G' becomes significantly greater than G", and the P-B-VH hydrogel underwent a solution to gel transition. In the strain mode, at a constant temperature of 37 ℃, G' was significantly greater than G"at low strain, and the hydrogel was in a structurally stable elastic solid phase. However, when the strain was greater than 25.12%, G" changed to greater than G', and the matrix network structure of the hydrogel was destroyed and was in an unstable viscoelastic liquid phase (**Figure 1G**). Subsequently, compression and tensile tests were conducted. As illustrated in **Figure 1H**, the hydrogel exhibited a compressive stress-strain curve characteristic of an elastic porous material, with a maximum compressive strength of 0.3 MPa. The uniaxial tensile test results presented in **Figure S2B** indicated that the hydrogel possessed appreciable tensile properties, achieving a tensile strength of approximately 471.7 kPa. The appropriate thermo-sensitive hydrogel should exhibit elastic solid properties after drug loading and filling into the bone defect site, and be able to maintain a stable and complete structure under complex *in vivo* mechanical conditions, which is a prerequisite to ensure its function 29. Our results indicates that the P-B-VH hydrogel has good thermosensitive gelation characteristics and structural stability.

In addition to bulk mechanical performance, strong adhesion between the material and host bone tissue is critical for ensuring functional efficacy. The robust interfacial bonding can effectively prevent hydrogel displacement *in vivo* 30. As shown in **Figure 1I**, the hydrogel demonstrates effective adhesion to bone tissue, achieving a maximum interfacial adhesion strength of approximately 2.3 kPa (**Figure 1J**).

Biodegradability plays an increasingly important role in the research and development of orthopedic biomaterials. The degradation of materials can fully release the drug contained in their matrixes, thus promoting bone repair, and the materials can be replaced by regenerated bone after degradation, avoiding the secondary surgery 31. Therefore, the degradability and biosafety of P-B-VH were subsequently evaluated. The hydrogel was gradually degraded within 56 days after injection into the subcutaneous tissue of the rats, and HE staining of the skin tissue at the injection site showed that the hydrogel did not cause significant rejection and toxic reactions in the local area (**Figure S3A-B**). Furthermore, HE staining of other systemic organs including heart, liver, spleen and kidney also showed similar results without obvious rejection and inflammation, demonstrating good biological safety of P-B-VH (**Figure S3C-F**).

The drug release from the P-B-VH hydrogel was subsequently evaluated. As shown in **Figure 1K**, both drugs exhibited sustained release over a period of 42 days; however, the release rate and peak concentration of vancomycin were significantly higher than those of BMP-2 peptide. Vancomycin released from P-B-VH followed a biphasic pattern: an initial burst release followed by sustained release. According to the release profile, 42.4±0.7% of vancomycin was released in the first 4 days, 61.4±0.7% in the first 14 days, and then the release rate slowed down significantly, gradually releasing to 81.0±1.9% of the total amount over a period of 42 days. For BMP-2 peptide, during the initial 14 days, only 9.80±0.50% of the peptide was released, followed by a phase of sustained and gradual release, reaching a cumulative release of 60.31±1.80% by day 32. Subsequently, the release rate of the BMP-2 peptide markedly decreased and entered a plateau phase, with a cumulative release amount of 71.18±1.96% observed on day 42. This release profile is consistent with the expectation of the treatment of infectious bone defects, that is, antibiotics need to rapidly reach effective concentrations to play an anti-infective role in the initial stage and BMP-2 exhibits a delayed release profile, with its peak concentration occurring in the subsequent second stage, thereby facilitating bone growth under the condition that infection has been adequately controlled 32. The release of vancomycin from P-B-VH mainly depends on two modes. In the early stage, the degradation rate of the hydrogel is slow, and the release of vancomycin is dominated by free diffusion. The drug located in the surface layer is released to the external environment faster and easier, while the free diffusion rate of the drug located in the interior is slower. In the later stage, the degradation rate of the hydrogel is significantly accelerated, and the drug release becomes dominated by material degradation, and the internal drug can be released to the external environment through a faster rate 22, 24. Finally, when the hydrogel was basically degraded around 56 days, the drug releasing was completed. The BMP-2 peptide was primarily released into the surrounding environment through the degradation of the hydrogel. Combination with the analysis of the degradation profile of the hydrogel showed that the release rate of the BMP-2 peptide increased with the increase of the degradation rate. Therefore, the drug release profile of P-B-VH hydrogel makes it a suitable biomaterial for the single-stage treatment of infected bone defects. It can rapidly release vancomycin in the initial stage to control the infection state. After the infection is well controlled, the BMP-2 peptide grafted by the hydrogel plays a bone-promoting role by acting with the BMSC *in situ* of the bone defect.

### Enhanced cell viability and proliferation

Stem cell antigen profiling was firstly performed on the isolated BMSCs. As shown in **Figure S4**, the BMSCs used in this study exhibit high expression of CD90 and absence of CD34, consistent with the established immunophenotypic characteristics of mesenchymal stem cells.

**Figure 2:**
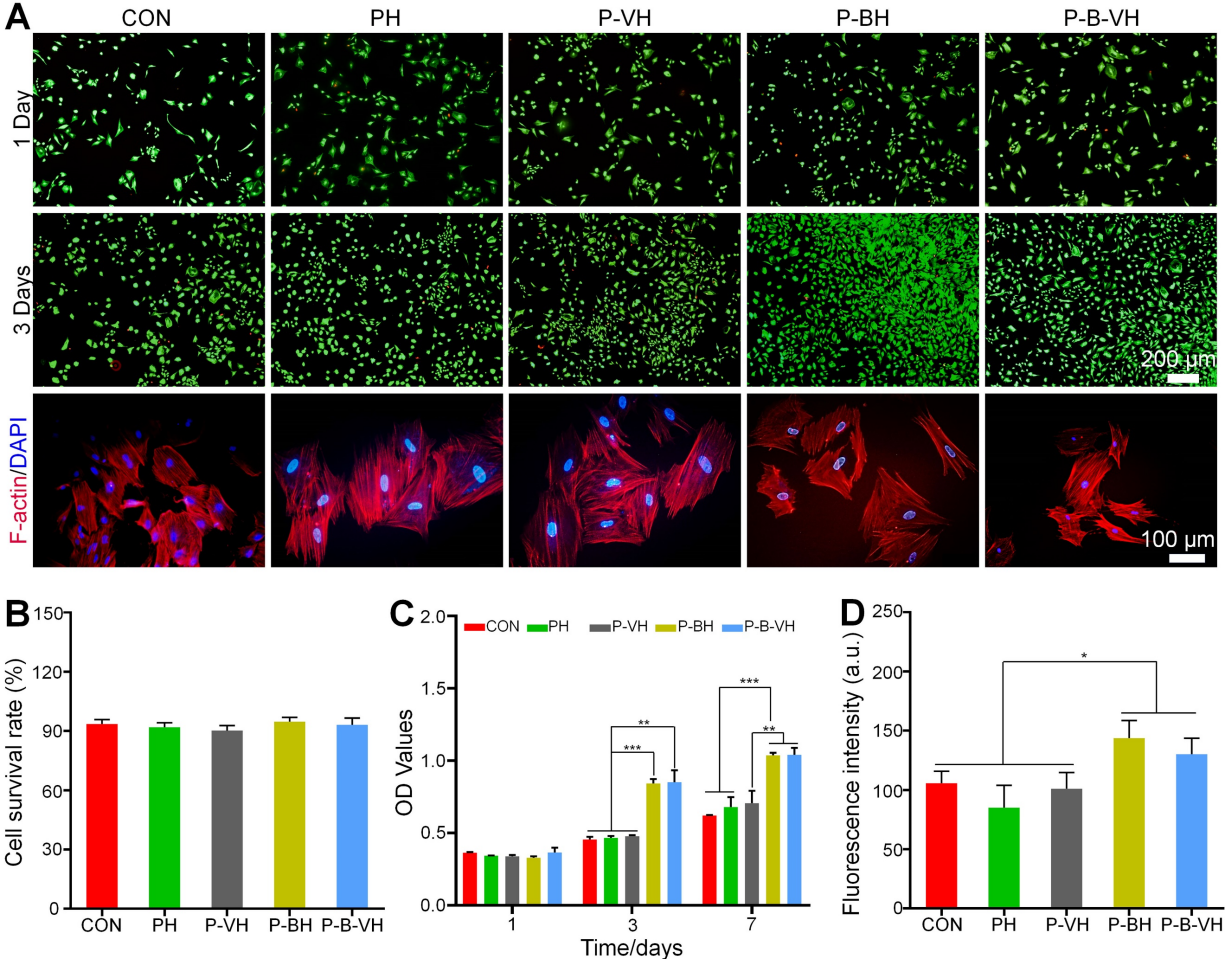
Effects of functionalized hydrogels on the proliferation and morphology of BMSCs: A. Calcein-AM/PI staining of BMSCs co-cultured with each group for 1 and 3 days and F-actin protein staining of the cytoskeleton after 3 days of co-culture; B. Cell survival rate analysis based on calcein-AM/PI staining; C. CCK-8 assay of BMSCs co-cultured with each group for 1, 3, and 7 days; D. Quantitative analysis of fluorescence intensity based on F-actin protein staining of the cytoskeleton; *p < 0.05, **p < 0.01, ***p < 0.001.

To examine the biocompatibility of the hydrogels, the Calcein-AM/PI experiment was performed. As shown in the **Figure 2A**, Calcine-AM was used to stain living cells, which resulted in green images under fluorescence microscope. Propidium lodide (PI) was used to mark dead cells, which resulted in red fluorescence. There were no obvious dead cells at day 1 and day 3 and the cell number augment gradually with the culture time in all groups. In addition, semi-quantitative analysis showed that cell viability was greater than 90% in each group, and there was no significant difference between the groups (**Figure 2B**). These results suggested that the hydrogel had good biocompatibility.

CCK-8 assay was further conducted to quantitatively evaluate the effects of the P-B-VH on cell proliferation. In **Figure 2C**, All the cells showed good proliferation activity within 7 days. The P-BH and P-B-VH groups grafted with BMP-2 peptide showed better cell proliferation than the other three groups on days 3 and 7. There were no significant differences between CON, PH and P-VH groups at both time points. This result confirmed that the grafting of BMP-2 brings functionality to the engineered hydrogel that facilitates BMSC proliferation. This is consistent with previous findings that osteogenic peptide derived from the knuckle epitopes of BMP-2 could promote BMSC proliferation by binding to BMP receptor type II 24, 33. Our results also confirmed that BMP-2 peptide retained good activity after hydrogel synthesis.

The morphological analysis of BMSC in each group was performed by rhodamine-labeled phalloidin staining. F-actin protein is the main component of cytoskeleton, which plays an important regulatory role in the early osteogenic differentiation, cell function and maturation of BMSC 34. As shown in **Figure 2A**, red-stained F-actin protein and blue-stained nuclei were observed in all groups. Semi-quantitative analysis further showed that the mean fluorescence intensity of F-actin in P-BH and P-B-VH groups increased, which was significantly higher than that in CON, PH and P-VH groups (**Figure 2D**). Previous studies have shown that cytoskeletal protein aggregation is critical for the differentiation and function of bone repair related cells 35. The spreading state of osteoprogenitor cells and the expression of cytoskeletal proteins are related to osteoblast differentiation. At the early stage of osteogenic differentiation, the morphology of the cells was significantly more spread, and the formation of actin microfilaments in the cytoplasm was significantly increased 36. Our results showed that the interaction between P-B-VH hydrogel and BMSC make the BMSC in a favorable condition for osteogenic differentiation.

### *In vitro* antimicrobial activity of the engineered hydrogel

**Figure 3:**
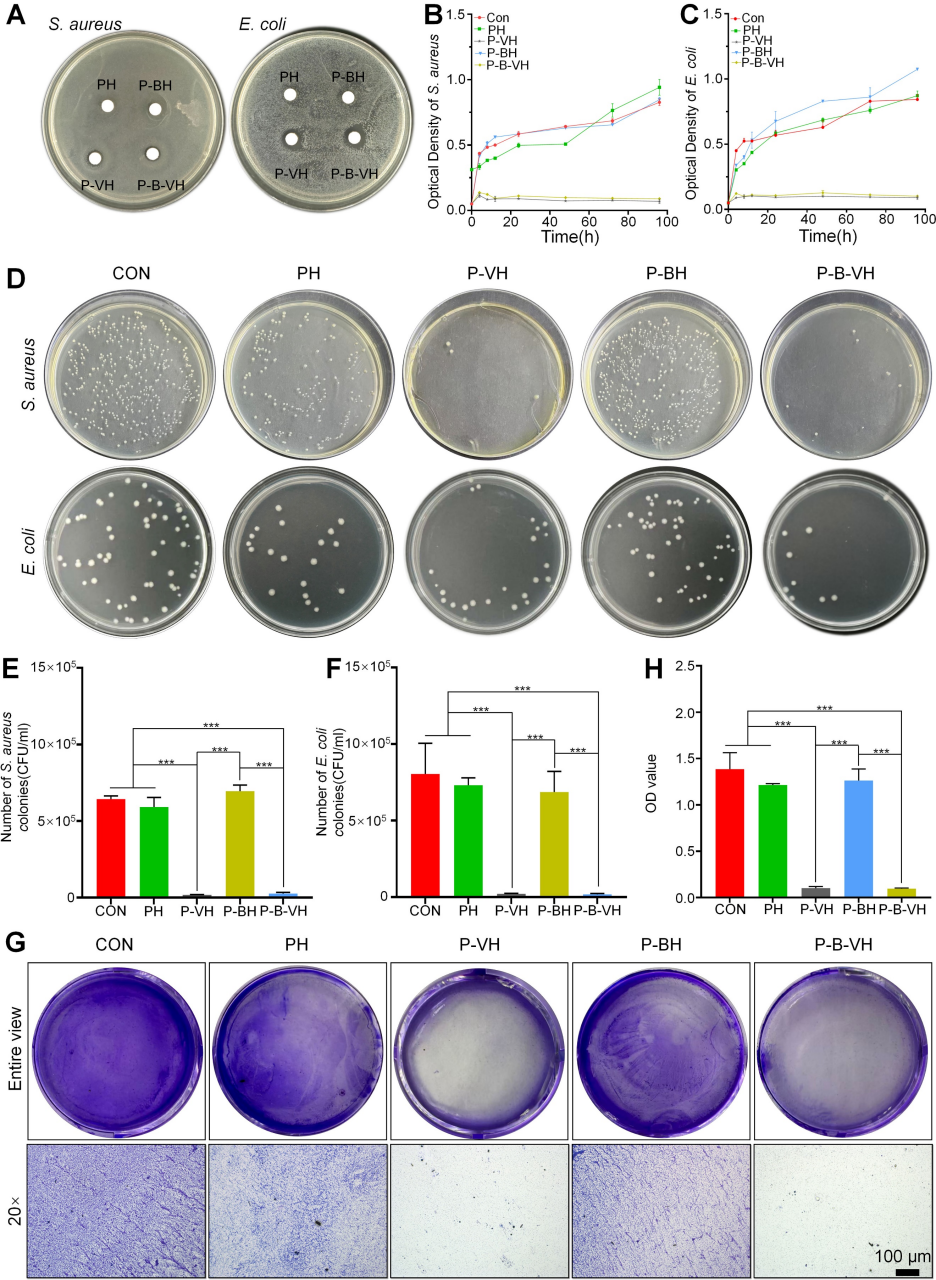
*In vitro* antibacterial function of P-B-VH hydrogel: A. Antibacterial zone test of *S. aureus* and *E. coli* co-cultured with each group of materials; B-C. Bacterial proliferation curves of *S. aureus* (B) and *E. coli* (C) co-cultured with each group; D. Bacterial plating test of *S. aureus* and *E. coli* co-cultured with each group for 24 h; E-F. Colony counts of *S. aureus* (E) and *E. coli* (F)in each group based on the bacterial plating test; G-H. Effect of each material on biofilm formation (G) and semi-quantitative analysis of biofilm content (H); *p < 0.05, **p < 0.01, ***p < 0.001.

Gram-negative *E. coli* and Gram-positive *S. aureus* bacteria were utilized as representative microorganisms to evaluate the antibacterial capabilities of P-B-VH. As shown in **Figure 3A**, after 24 h of incubation, no inhibition zones were observed in the PH and P-BH groups, while the P-VH and P-B-VH groups with vancomycin both showed superior antibacterial properties with significant inhibition zone against* E. coli* and *S. aureus* around the hydrogel under the same conditions. The sizes of inhibition zone against* E. coli* and *S. aureus* were 25.6±0.2 mm and 18.3±0.5 mm in P-VH group and 28.2±0.5 mm and 17.6±0.5 mm in P-B-VH group, respectively. There was no significant difference in inhibition zone sizes for both *E. coli* and *S. aureus* between P-VH and P-B-VH groups.

To further quantify the antibacterial activity of P-B-VH hydrogel, bacterial proliferation profile and plate counting assay were performed. **Figure 3B and C** illustrated the bacterial proliferation profile of *S. aureus* and *E. coli* during 96 h of co-culture with CON, PH, P-BH, P-VH, and P-B-VH groups, respectively. In the first 24 h, the proliferation of both bacteria in the CON, PH and P-BH groups was rapid, and then entered a relatively flat stage. However, the proliferation of both bacteria in P-VH and P-B-VH groups was significantly inhibited after 6 h. This trend was further confirmed by plate counting results at 24 h, which showed that the colony numbers of *S. aureus* and* E. coli* in P-VH and P-B-VH groups were significantly lower than those in the other three groups (**Figure 3D**). The results of quantitative analysis showed that CON, PH and P-BH groups had similar colony counts (*S. aureus* > 6×10^5^ CFU and E*. coli* > 8×10^5^ CFU ), which were significantly higher than those of P-VH group (0.2±0.02×10^5^ CFU and 0.2±0.03×10^5^ CFU) and P-B-VH group (0.3±0.07×10^5^ CFU and 0.2±0.06×10^5^ CFU) (**Figure 3 E, F**).

The ability of infectious bacteria to attach to the surface of bone or graft to form biofilms makes them highly resistant to antimicrobial agents and evade detection by the immune system, leading to a chronic infection state 37. Therefore, the inhibitory effect of P-B-VH on biofilms was investigated. As illustrated in **Figure 3G and 3H**, the formation of purple-stained biofilms in the P-VH and P-B-VH groups, which contained vancomycin, was markedly reduced compared to that in the CON, PH, and P-BH groups, indicating a significant inhibitory effect on biofilm development.

These findings indicate that the vancomycin incorporated within the network matrix of P-B-VH can be released into the surrounding environment to exert sustained and effective antibacterial function.

### Enhanced osteogenic differentiation *in vitro*

**Figure 4:**
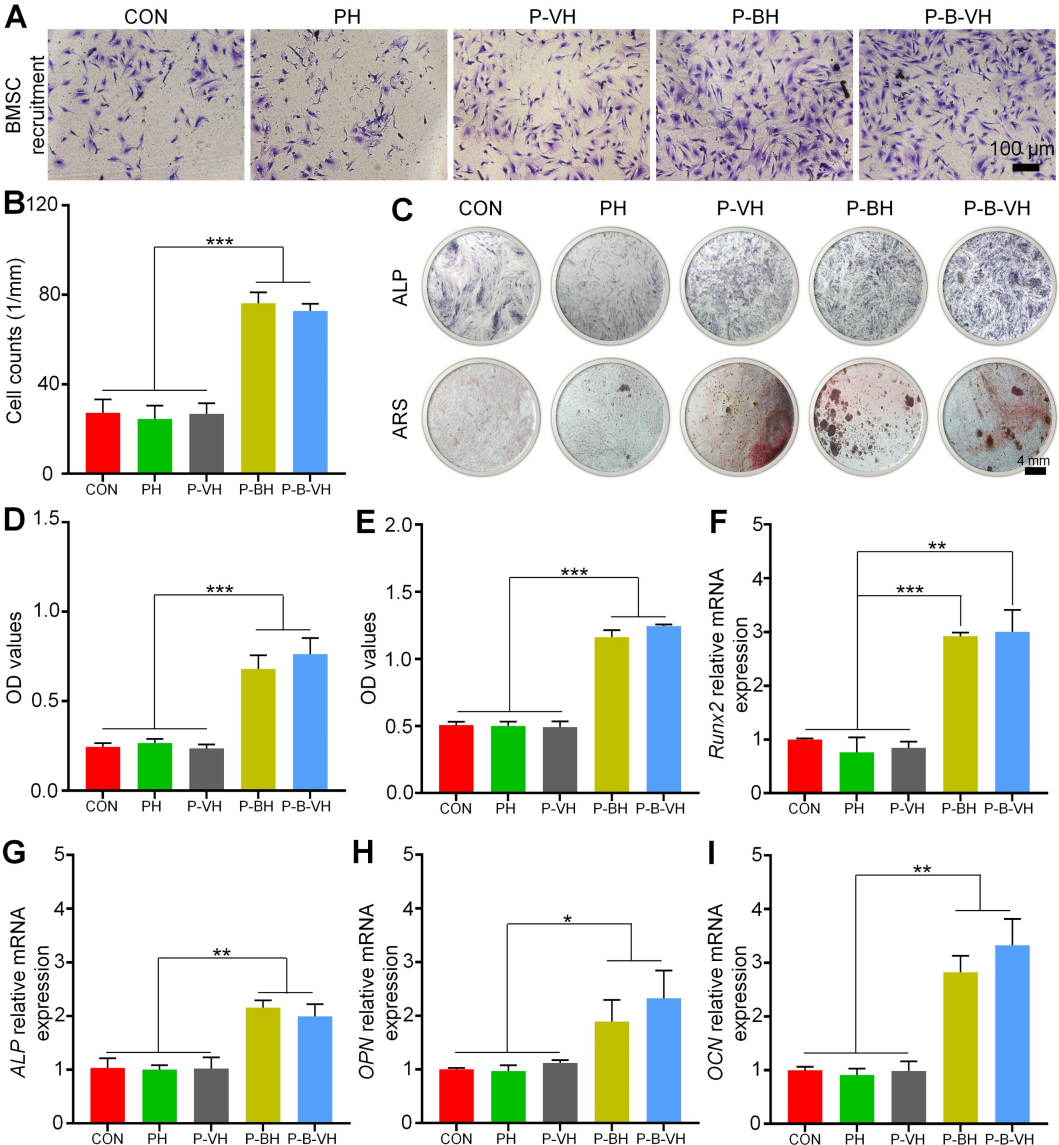
*In vitro* osteogenic functionality of P-B-VH hydrogel: A. Evaluation of cell migration after co-culture of BMSC with each group for 24 hours by Transwell assay; B. Quantitative analysis based on the results of the Transwell experiment; C. ALP staining of BMSCs co-cultured with each group for 7 days and ARS staining after 14 days; D. ALP enzyme activity analysis of BMSCs co-cultured with each group for 7 days; E. Semi-quantitative analysis of calcium nodules based on ARS staining; F-I. RT-qPCR detection of osteogenic-related gene *Runx2* (F), *ALP*(G), *OPN*(H) and* OCN* (I) expression levels of BMSCs co-cultured with each group for 7 days; *p < 0.05, **p < 0.01, ***p < 0.001.

In the process of bone formation, BMSC and osteoblasts need to first accurately migrate to the injured site to initiate osteogenic differentiation and bone repair 38. The specific capacity of P-B-VH hydrogel to induce BMSC migration *in vitro* was first evaluated. As shown in **Figure 4A-B**, the higher amount of BMSCs stained purple in the P-BH and P-B-VH groups, which contained the BMP-2 peptide, exhibited significantly greater cell migration rate compared to those in the other three groups. No significant difference was observed between the P-BH and P-B-VH groups.

ALP is one of the main markers of osteogenic differentiation of BMSC, which is secreted by osteoblasts in the form of vesicles and participates in the maturation and mineralization of extracellular matrix. **Figure 4C** demonstrated representative images of ALP staining after 7 days of co-culture of hydrogel and BMSC in each group. The end product, which was stained purple, was significantly higher in the P-B-VH and P-BH groups than in the CON, PH, and P-VH groups, indicating a higher ALP content. Further quantitative analysis of ALP enzyme activity showed the same trend, with the P-B-VH and P-BH groups having similar ALP enzyme activity and significantly higher than the other three groups (**Figure 4D**).

Calcium deposition and mineralization of extracellular matrix are important markers for the maturation of BMSC osteogenic differentiation 39. ARS was performed to evaluate calcium nodules secreted into the extracellular space. Macroscopic images and semi-quantitative analysis showed that P-BH and P-B-VH groups had significant calcium deposition, which has significantly higher mineralization level of BMSC than that of CON, PH and P-VH groups (**Figure 4C, E**).

Furthermore, RT-qPCR experiment was performed to analysis the osteogenic genes expression levels. *Runx2* is a key gene in the early stage of osteogenic differentiation, which can promote the expression of bone matrix proteins and the maturation of osteoblasts 40. After 7 days of osteogenic induction, the P-B-VH (3.00±0.33) and P-BH (2.92±0.05) groups showed higher *Runx2* relative expression levels than the CON (1.00±0.02), PH (0.76±0.23) and P-VH (0.84±0.10) groups** (Figure 4F)**. *ALP*, *OPN*, and *OCN* are well-established biomarkers representing the extracellular matrix mineralization stage during osteogenic differentiation 41. The expression levels of these three genes exhibited a consistent trend, whereby the P-BH and P-B-VH groups showed comparable expression, which was significantly higher than that observed in the other three groups on day 7 of osteogenic induction (**Figure 4G-I**).

**Figure 5:**
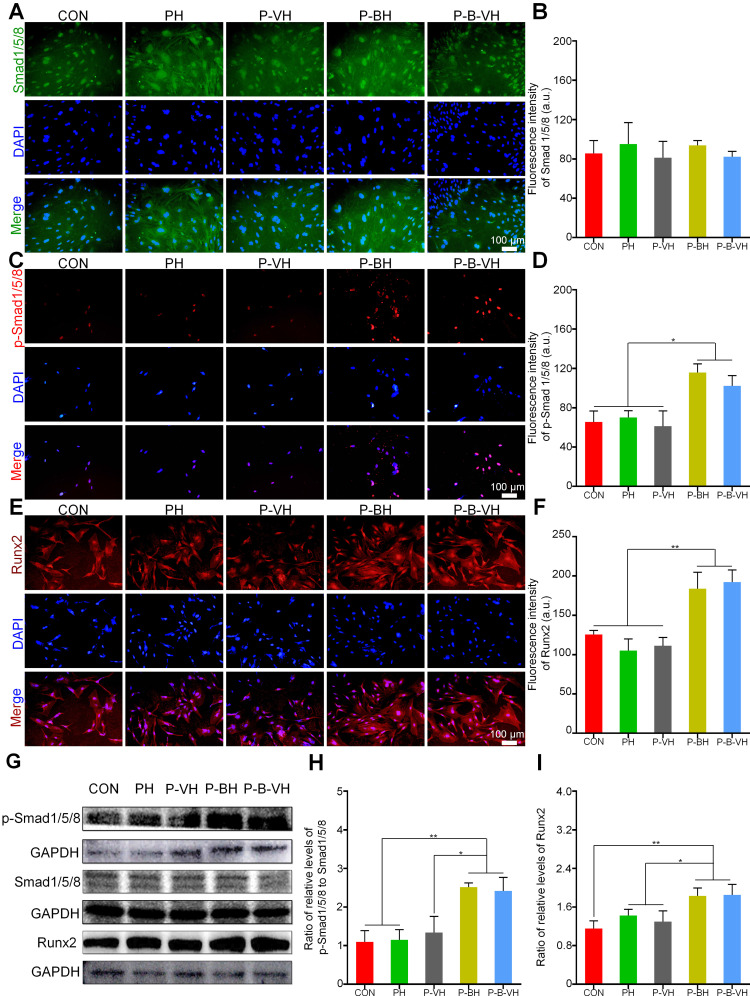
Verification of the molecular mechanism of P-B-VH promoting osteogenesis: A-F. Immunofluorescence staining of Smad1/5/8 (A), p-Smad1/5/8 protein (C) and Runx2 (E), and fluorescence intensity analysis based on staining results (B, D, F) after BMSC culturing in each group for 3 days; G: Western Blot detection of p-Smad1/5/8, Smad1/5/8 and Runx2 proteins after 3 days of BMSC culture in each group; H-I: Semi-quantitative analysis of gray values based on Western Blot detection results;*p < 0.05, **p < 0.01.

The knuckle epitope of BMP-2 has been confirmed to bind to BMP-binding receptor type II on the cell surface, and then activate downstream Smad/Runx2 signal pathway to regulate osteogenic differentiation 24. After binding to the BMP-binding receptor type II, the signaling cascade activates downstream Smad1/5/8 protein phosphorylation. The p-Smad1/5/8 subsequently forms a complex with Smad 4 and is translocated into the nucleus. This complex interacts with specific DNA sequences to upregulate the expression of Runx2 and its downstream osteogenesis-related genes, thereby regulating osteogenic differentiation 42, 43. As shown in **Figure 5A-B**, no significant differences in total Smad protein expression levels were observed among the groups. However, the P-B-VH and P-BH group exhibited significantly higher fluorescence intensity of p-Smad1/5/8 and Runx2 proteins compared to the CON, PH, and P-VH groups (**Figure 5 C-F**). Further Western blot analysis demonstrated that the protein expression levels of p-Smad1/5/8 and its downstream target Runx2 were significantly elevated in the P-B-VH and P-BH groups compared to the CON, PH, and P-VH groups, whereas the total Smad1/5/8 protein levels remained comparable across all groups (**Figure 5G**). Subsequent quantitative analysis of band intensities based on gray values revealed that the ratios of p-Smad1/5/8 to total Smad1/5/8 were markedly higher in the P-B-VH and P-BH groups than in the other three groups (**Figure 5H**), indicating enhanced phosphorylation and activation of Smad1/5/8 in these treatment groups. A similar trend was observed for the expression levels of the downstream protein Runx2 (**Figure 5I**). These findings confirmed that the P-B-VH hydrogel promoted osteogenic differentiation primarily through activation of the Smad/Runx2 signaling pathway.

Taken together, a functional osteogenic material was developed by grafting a peptide derived from the BMP-2 knuckle epitope onto the mPEG-poly (amino acid) hydrogel. Upon co-culturing, the Smad/Runx2 signaling pathway in BMSCs was activated via sustained release of the BMP-2-derived peptide from P-B-VH, thereby enhancing early osteogenic differentiation and extracellular matrix mineralization. Our *in vitro* results confirmed the construction of P-B-VH hydrogel with rapid antibacterial and stable osteopromoting properties for the treatment of infected bone defects.

### Effective infection control at the defect site

Encouraged by the excellent cytocompatibility, rapid antibacterial activity, and bone-promoting activity demonstrated by the P-B-VH hydrogel *in vitro*, we further evaluated its potential for treating infected bone defects *in vivo*. Specifically, an infected rabbit radius defect model was firstly established and assessed. As illustrated in **Figure S5 A-B**, from day 4 to day 14 post-modeling, the white blood cell and neutrophil counts in the rabbits of the model group were significantly elevated compared to those in the normal control group. At the week 2, the model group exhibited pronounced abscess formation and sinus tract development in the mid-radius region** (Figure S5 C)**. X-ray imaging revealed marked local soft tissue swelling **(Figure S5 D)**. Giemsa staining indicated extensive bacterial colonization in the model group. Furthermore, HE staining demonstrated significant infiltration of inflammatory cells in the affected area, with partial destruction of the trabecular structure **(Figure S5 E-F)**. Based on these findings, the rabbit infected radius defect model was successfully established.

**Figure 6:**
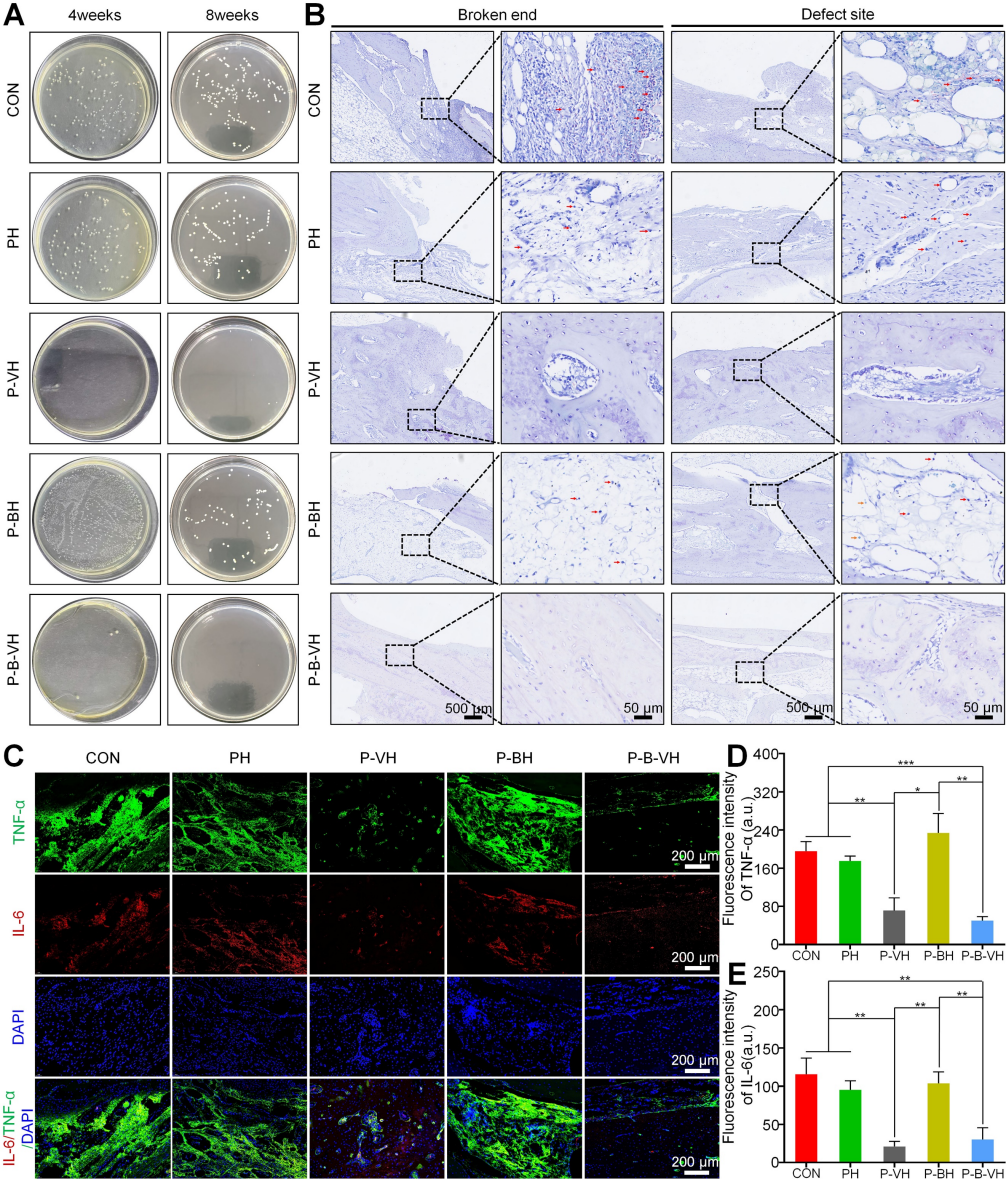
*In vivo* antibacterial function of P-B-VH hydrogel: A. Bacterial culture at the defect site at week 4 and 8 post-operation; B. Giemsa staining of bacteria at the broken ends and defect sites at 8 weeks post-operation (The red arrow marked area in the figure represents the stained bacteria); C-E. Immunofluorescence staining of the inflammatory factors TNF-α and IL-6 in the defect site at 4 weeks after surgery (C) and semi-quantitative analysis of the fluorescence intensity of TNF-α (D) and IL-6 (E) based on the immunofluorescence results.

After confirming the successful establishment of the infectious bone defect model, local debridement was performed, followed by the transplantation of the hydrogel to the affected area. Initial assessment focused on infection control. Radiographic analysis revealed that by week 4, a small amount of new bone regeneration had already occurred in the P-B-VH group. Consequently, samples were collected at weeks 4 and 8 for further infection evaluation. As illustrated in **Figure 6A**, no bacterial colonies were detected at the defect site in the P-B-VH and P-VH groups at week 4 post-surgery. In contrast, substantial bacterial growth was observed in the CON, PH, and P-BH groups. At week 8, the results of bacterial plating from the defect sites in each group continued to exhibit the same trend, indicating that both the P-B-VH group and the P-VH group achieved effective antibacterial treatment with no recurrence of infection. Giemsa staining of the defect site corroborated these findings, demonstrating no residual bacterial colonization in the P-B-VH and P-VH groups at weeks 8, whereas significant bacterial colonization persisted in the remaining three groups** (Figure 6B)**.

Infection can induce a prolonged hyperinflammatory state within bone defects. The sustained presence of various pro-inflammatory cytokines negatively affects osteogenic differentiation, thereby impeding the bone repair process. Among these cytokines, TNF-α and IL-6 are representative local pro-inflammatory mediators following bone infection 3, 4. As illustrated in **Figure 6C-E**, the P-B-VH and P-VH groups exhibited significantly reduced fluorescence intensities of TNF-α and IL-6 compared to the CON, PH, and P-BH groups. These findings indicated that both P-B-VH and P-VH can effectively manage infection through sustained antibiotic release, thereby suppressing excessive local inflammation and promoting the formation of an immunological microenvironment favorable to bone regeneration.

Additionally, at week 8, HE staining confirmed a marked reduction in inflammatory cell infiltration in the P-B-VH and P-VH groups compared to the CON, PH, and P-BH groups, along with evidence of new bone formation** (Figure 8)**. These results indicate that infection in the P-B-VH group was effectively controlled.

### Promoting the bone reconstruction of infected bone defects

**Figure 7:**
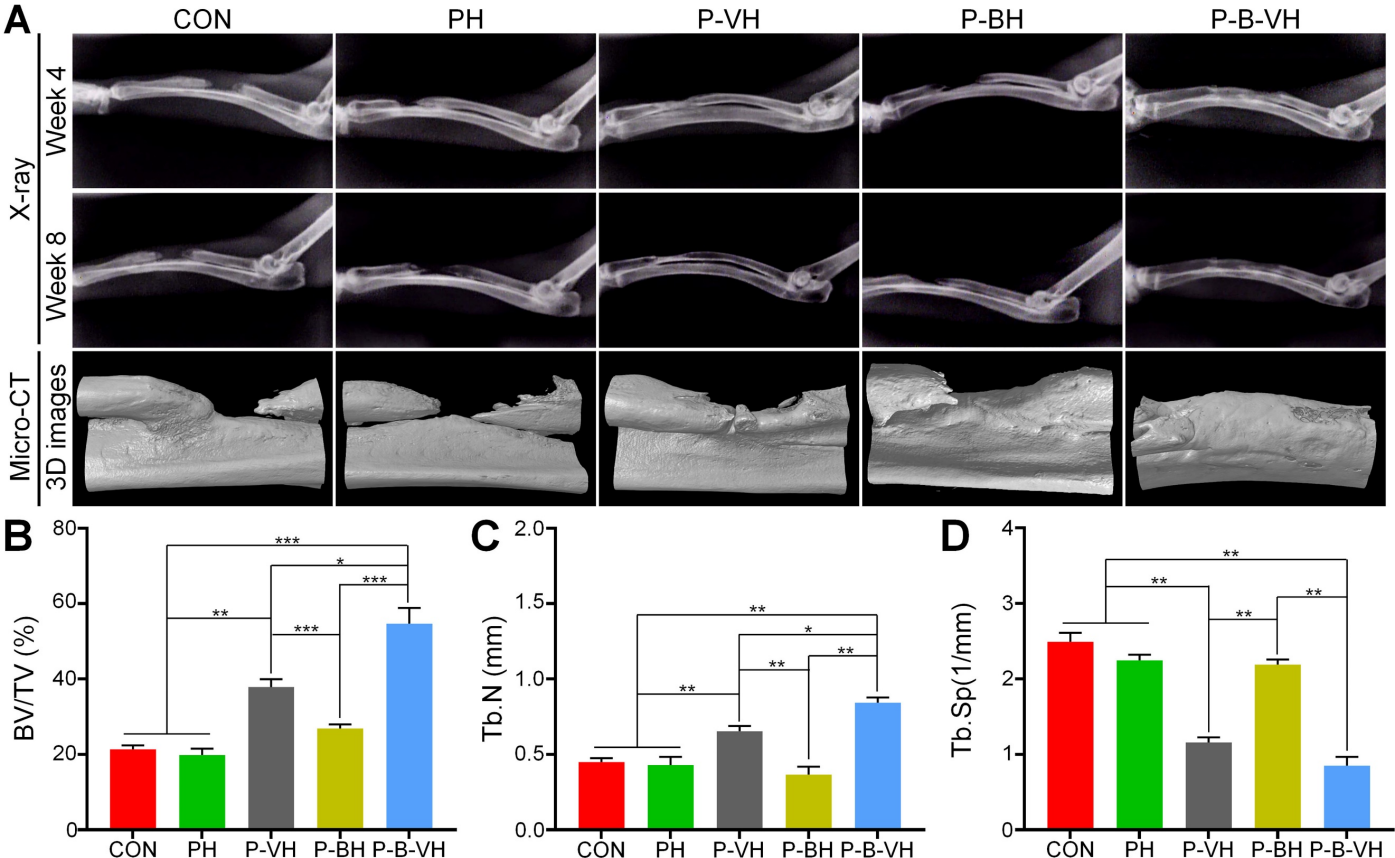
Imaging evaluation of P-B-VH in the repair of infectious bone defects: A. Representative images of postoperative X-ray images and Micro-CT reconstructions; B-D. Data analysis of BV/TV (B), Tb.N (C), and Tb.Sp (D) within the region of interest based on Micro-CT; *p < 0.05, **p < 0.01, ***p < 0.001.

After the hydrogel was implanted into the infected bone defect, dynamic and regular X-ray examinations revealed that a significant amount of callus formation was observed locally in the P-B-VH group at the week 4 post-surgery. In contrast, only a minimal amount of callus formation was detected in the P-VH group, while no apparent callus formation was observed in the other groups. By the week 8, the P-B-VH group demonstrated excellent local bone regeneration and repair. The P-VH group exhibited callus formation; however, the defect remained evident. Meanwhile, the CON, PH, and P-BH groups showed no new callus formation and exhibited signs of local bone destruction (**Figure 7A**).

Radial samples from each group were harvested for Micro-CT scanning and analysis at 8 weeks postoperatively. **Figure 7A** presents the 3D reconstructed representative images of the scan results for each group at the week 8. In the P-B-VH group, new bone tissue had successfully filled the defect site and bridged the fractured ends. Although local new bone formation was observed in the fractured ends of the P-VH group, the overall regenerated bone mass was limited, and the defect remained unfilled. No evidence of local new bone formation was detected in the PH and P-BH groups; instead, the marrow cavity of the fractured ends was closed, and signs of bone destruction, absorption, and nonunion were evident. Statistical analysis revealed that the BV/TV ratio in the P-B-VH group (54.7±4.2%) was significantly higher than in the other four groups. Additionally, the BV/TV ratio in the P-VH group (37.9±2.1%) was significantly greater than in the CON (21.3±1.1%), PH (19.8±1.7%), and P-BH (26.9±1.1%) groups, with no significant differences among these three groups (**Figure 7B**). Furthermore, the P-B-VH group demonstrated the highest trabecular number, as well as the lowest trabecular separation. The P-VH group also exhibited superior trends compared to the CON, PH, and P-BH groups (**Figure 7C-D**).

**Figure 8:**
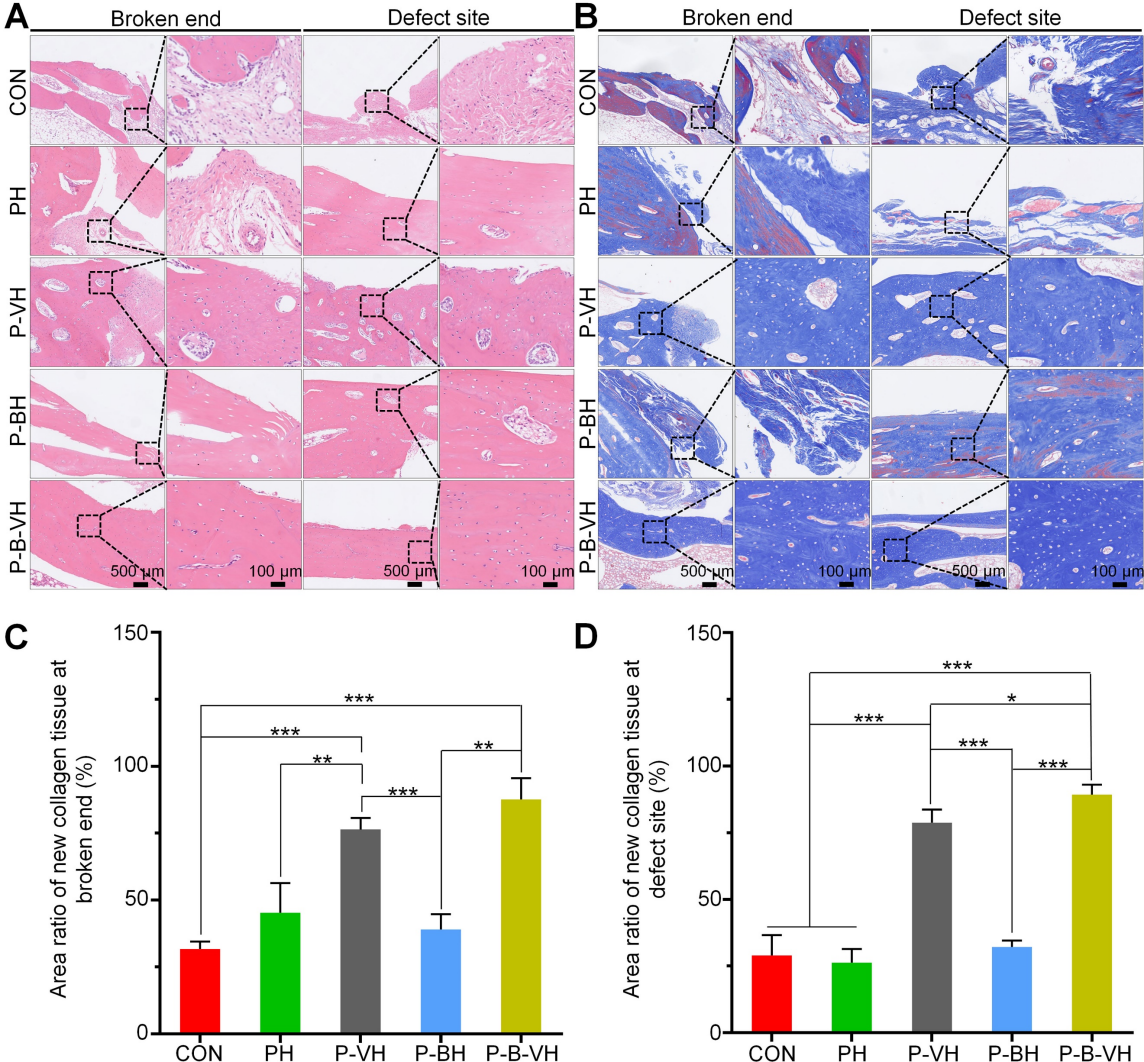
Histological assessment of P-B-VH in the repair of infectious bone defects: A. HE staining of the broken ends and defect site at 8 weeks post-operation; B. Masson staining of the broken ends and defect site at 8 weeks post-operation; C-D. Proportion of new collagen in the broken ends (C) and defect site (D) based on Masson staining; *p < 0.05, **p < 0.01, ***p < 0.001.

HE and Masson staining were subsequently performed for the histological evaluation of local bone repair. As illustrated in **Figure 8A**, the broken ends in the CON, PH, and P-BH groups exhibited predominantly old bone tissue, with no evident signs of new bone formation. Additionally, the marrow cavity was sealed, leading to bone destruction and resorption. In contrast, a small amount of new bone tissue was observed at the broken end in the P-VH group, while a substantial amount of new bone tissue was detected in the P-B-VH group. The staining results from the defect site revealed a similar trend. Specifically, inflammatory connective tissue was locally filled in the defect site of the CON, PH, and P-BH groups without new bone. In the P-VH group, a minor presence of new bone tissue was noted. Notably, in the P-B-VH group, prominent bone regeneration was observed, with newly formed bone bridging the defect site.

In Masson staining, the collagen fibers in mature bone tissue predominantly appeared red, whereas those in newly formed bone tissue were primarily blue. As depicted in **Figure 8B**, the CON, PH, and P-BH groups predominantly displayed red-stained mature collagen fibers, with only a limited area of blue-stained new collagen fibers, which is orderly arranged old bone tissue. In contrast, the P-VH group showed a significant increase in the area of blue-stained new collagen fibers, while the P-B-VH group predominantly exhibited blue-stained new collagen fibers with minimal red-stained mature collagen fibers, and the collagen fibers were irregularly arranged, indicating the presence of substantial new bone tissue. Subsequent semi-quantitative analysis confirmed this trend, revealing that the proportion of new collagen fibers at the fracture ends in the P-B-VH group was significantly higher than that in the CON, PH, P-BH and P-VH group (**Figure 8C**). The staining results and semi-quantitative analysis of the defect site also demonstrated a consistent trend: the CON, PH, and P-BH groups predominantly exhibited orderly red-stained ulna collagen fibers; the P-VH group showed a small amount of new radius tissue; and the P-B-VH group exhibited a large amount of primarily blue-stained new bone tissue at the defect site (**Figure 8 B, D**).

**Figure 9:**
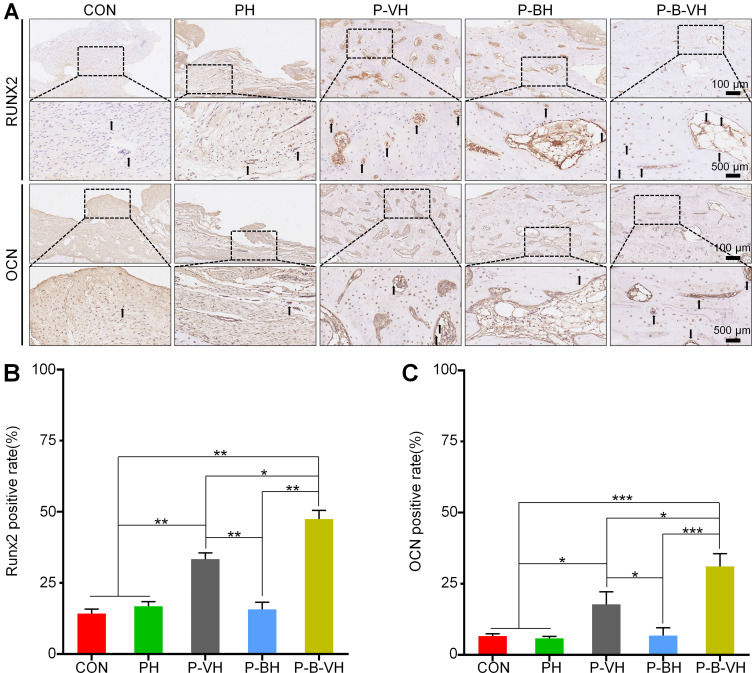
Effects of P-B-VH hydrogel on osteogenic proteins *in vivo*: A. Immunohistochemical staining of Runx2 and OCN at the defect site 8 weeks after surgery; B-C. Semi-quantitative analysis of the positive rates of Runx2 (B) and OCN (C) based on immunohistochemical staining (The black arrows represent staining positive cells); *p < 0.05, **p < 0.01, ***p < 0.001.

To further investigate the specific mechanism by which P-B-VH accelerates bone repair in infected bone defects, the expression levels of osteogenesis-related proteins Runx2 and OCN in local tissues were assessed. As depicted in **Figure 9A**, a substantial number of Runx2- and OCN-positive cells were observed at the fracture ends and defect sites in the P-B-VH group. In the P-VH group, positive cells were detected at the fracture ends but were scarce at the defect sites. The CON, PH, and P-BH groups exhibited only a limited number of Runx2- and OCN-positive cells at both the fracture ends and defect sites. Subsequently, semi-quantitative analysis further corroborated that the number of Runx2- and OCN-positive cells at the fracture ends and defect sites in the P-B-VH group was significantly higher than in the other four groups. Additionally, the number of positive cells in the P-VH group was also markedly greater than in the CON, PH, and P-BH groups (**Figure 9 B-C**).

The treatment of infectious bone defects represents a significant challenge in orthopaedic trauma, as it necessitates addressing both infection and bone defect simultaneously 44. Among these challenges, the control of infection is the primary concern that must be addressed. Bacterial pathogens and the resultant chronic inflammatory state can directly suppress osteoblast function, induce osteoblast apoptosis, or cause cell necrosis by damaging the osteoblast cell membrane. Additionally, infection state can indirectly alter the osteogenic microenvironment through the induction of extracellular matrix degradation and disruption of the osteoblast/osteoclast balance 3, 4. However, local pathogens cannot be fully eradicated through debridement alone. Our findings demonstrated that in the CON, PH, and P-BH groups, which did not involve continuous local antibiotic release following debridement, the progression of infection could not be effectively controlled, and persisted up to week 8. In contrast, the P-VH and P-B-VH groups exhibited satisfactory control of infection due to the rapid and efficient early release of vancomycin from hydrogel. However, the repair of residual bone defects following infection control remains a critical challenge, as bone tissues are incapable of achieving complete regeneration without intervention 45. Although new bone formation is observed in the P-VH group, defective bone nonunion occurs. In contrast, the P-B-VH group leverages BMP-2 peptides to act on in-situ stem cells post-infection control, thereby promoting osteogenic differentiation. This mechanism accelerates bone repair and demonstrates the most efficacious therapeutic outcome.

Compared with the traditional protocol, P-B-VH exhibits dual functionalities of antibacterial activity and bone promotion, along with degradability. This avoids multiple surgical interventions and simultaneously addresses both infection and defect issues through a single-stage surgery. However, the optimal design of bifunctional materials should enable distinct functions at different stages, namely early infection control followed by subsequent bone promotion. Premature activation of the osteogenic function during the infection control phase may compromise overall efficacy 46-48. Our findings corroborated that the therapeutic efficacy of the P-BH group was not significantly different from that of the untreated control group, as osteogenesis therapy is unlikely to be effective in the absence of proper infection management. In this study, the P-B-VH hydrogel was engineered to incorporate vancomycin via physical blending and BMP-2 peptides through chemical grafting. This ensures rapid release of vancomycin during the early stage for infection control, while the BMP-2 peptide continues to interact with local BMSCs in the later stage to promote osteogenic differentiation. Consequently, this study presents a single-stage antibacterial-osteogenic sequential therapeutic strategy for infectious bone defects.

## Conclusions

In this study, a vancomycin-loaded and BMP-2 grafted mPEG-poly (Ala-Phe) hydrogel was developed for the single-stage treatment of infectious bone defects. Different loading methods impart distinct temporal characteristics to the antibacterial and osteogenic functions, leading to variations in their effectiveness timing. The engineered hydrogel exhibits antibacterial efficacy through the rapid release of vancomycin at an early stage to control the infection. Simultaneously, the hydrogel demonstrates excellent biocompatibility, and the grafted BMP-2 peptide subsequently supports the proliferation of *in situ* BMSC, promotes the expression of osteogenic genes and proteins, and thereby accelerates local bone repair in infected bone defects. Overall, the engineered hydrogel designed in this study holds significant clinical potential, offering a single-stage strategy for sequential infection control and osteogenic promotion in the treatment of infectious bone defects.

## Supplementary Material

Supplementary table of primer sequences (Table S1); ^1^H NMR spectra and mass spectrometry analysis of the synthesized L-Ala-NCA and L-Phe-NCA (Figure S1); evaluation of the pore size of the PH and P-B-VH hydrogel and the tensile strength assessment of P-B-VH hydrogel (Figure S2); evaluation of the biodegradability and histocompatibility of the P-B-VH hydrogel (Figure S3); immunofluorescence staining of stem cell marker proteins of BMSC (Figure S4); evaluation of the animal model of infectious bone defect (Figure S5).
