# Supplementary material for: Engineered Poly (amino acid) Hydrogel Synchronizes Sequential Infection Elimination and Osteogenic Activation for Single-stage Reconstruction of Infected Bone Defects
Source: Theranostics. 2026 Jan 1;16(6):3206–26. doi: 10.7150/thno.121585 (PMC12775935; doi:10.7150/thno.121585)
Supplement: Supplementary file 1 — Supplementary table of primer sequences (Table S1); 1H NMR spectra and mass spectrometry analysis of the synthesized L-Ala-NCA and L-Phe-NCA (Figure S1); evaluation of the pore size of the PH and P-B-VH hydrogel and the tensile strength assessment of P-B-VH hydrogel (Figure S2); evaluation of the biodegradability and histocompatibility of the P-B-VH hydrogel (Figure S3); immunofluorescence staining of stem cell marker proteins of BMSC (Figure S4); evaluation of the animal model of infectious bone defect (Figure S5). [file thnov16p3206s1.pdf]

Engineered poly (amino acid) hydrogel synchronizes sequential infection elimination and osteogenic activation for single-stage reconstruction of infected bone defects

Yutao Cui[a,1], Yuhang Tian[a,1], Minghan Dou[a], Shaorong Li[a], Yi Fan[a], Gan Wang[a], Chuangang Peng[a], Yanbing Wang[a], Dankai Wu[a,*]

[a]Orthopedic Medical Center, The Second Hospital of Jilin University, Changchun, China

[1] These authors contributed equally to this work.

*** Correspondence**

Dankai Wu,

wudk@jlu.edu.cn

Table S1 The primer sequences in this study

Gene subtype	Oligonucleotide Primers (5'-3')
Runx2	F: 5′-TCAGGCATGTCCCTCGGTAT-3′<br>R: 5′-TGGCAGGTAGGTATGGTAGTGG-3′
ALP	F: 5′-GCACTCCCACTTTGTCTGGA-3′<br>R: 5′-TCCTGTTCAGCTCGTACTGC-3′
OPN	F: 5′-CACCATGAGAATCGCCGT-3′<br>R: 5′-CGTGACTTTGGGTTTCTACGC-3′
OCN	F: 5′- CAGAGCGACAGCATGAGGG-3′<br>R: 5′- CCTCTGCCAGACTCTGCACC-3′
GAPDH	F: 5′- GTTCCACGGCACGGTCAAGG-3′<br>R: 5′- CCAGGGGGGCTAAGCAGTTG-3′

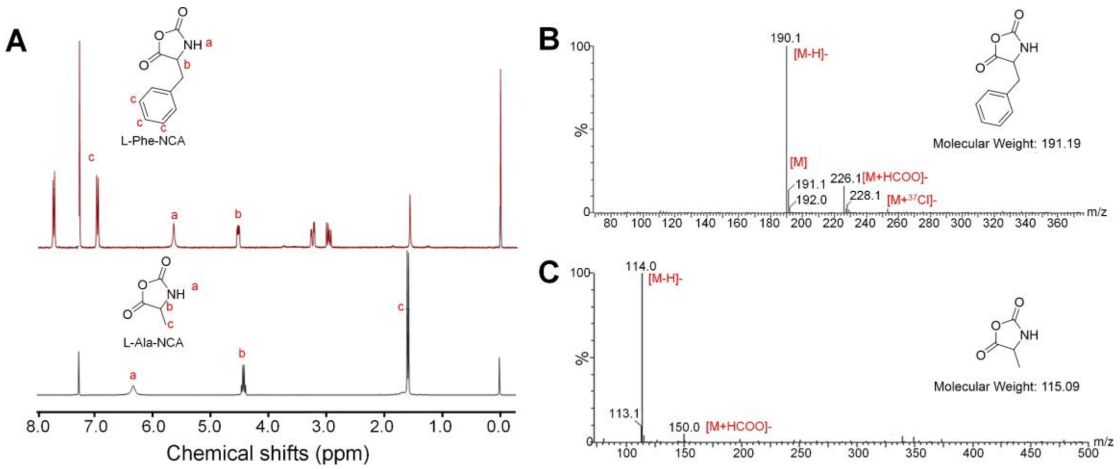

Figure S1. Characterization of L-Ala-NCA and L-Phe-NCA: A. ^{1}H NMR spectra of the synthesized L-Ala-NCA and L-Phe-NCA; B-C. Mass spectrometry analysis of the synthesized L-Ala-NCA (B) and L-Phe-NCA (C).

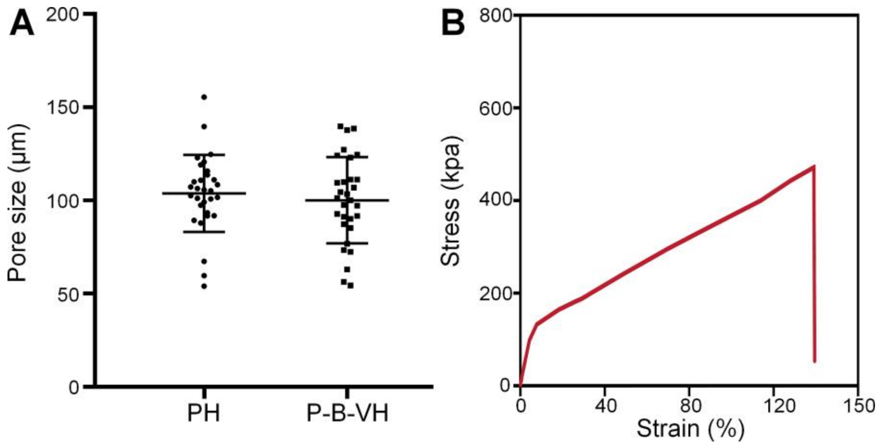

Figure S2. A. Evaluation of the pore size of the PH and P-B-VH hydrogel; B. Measurement of tensile strength of P-B-VH hydrogel.

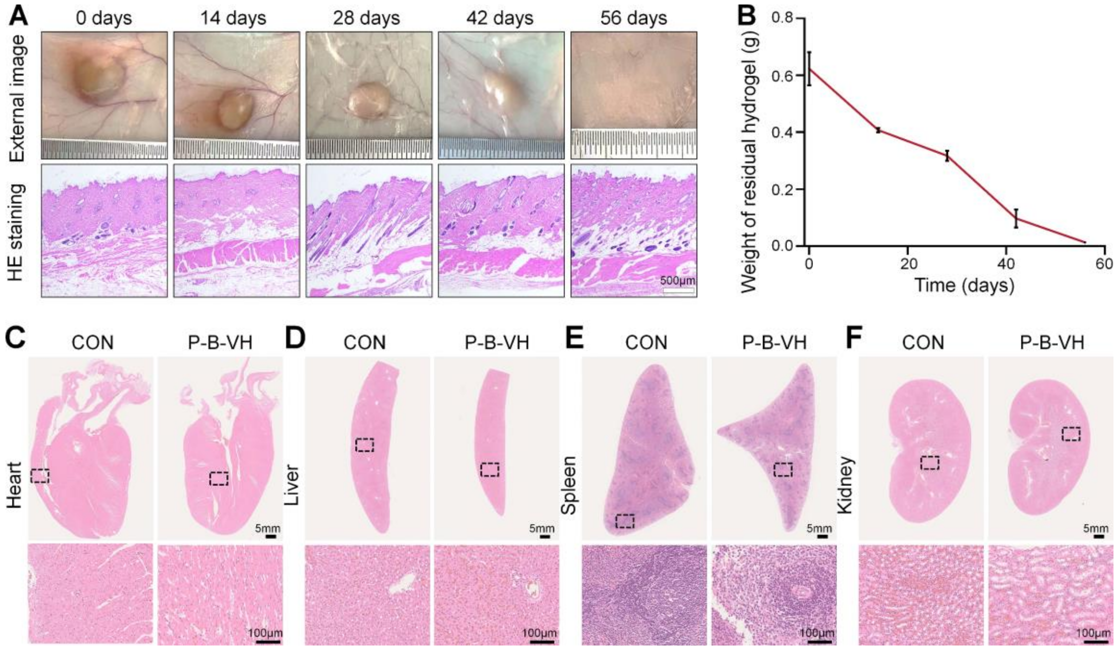

Figure S3. Evaluation of the biodegradability and histocompatibility of the P-B-VH hydrogel: A. Subcutaneous degradation of P-B-VH hydrogel and HE staining of the skin at the injection site; B. Quantitative analysis of the residual hydrogel weight of P-B-VH hydrogel after subcutaneous degradation; C-F. HE staining of the heart (C), liver (D), spleen (E) and kidney (F) after subcutaneous implantation of P-B-VH hydrogel in rats.

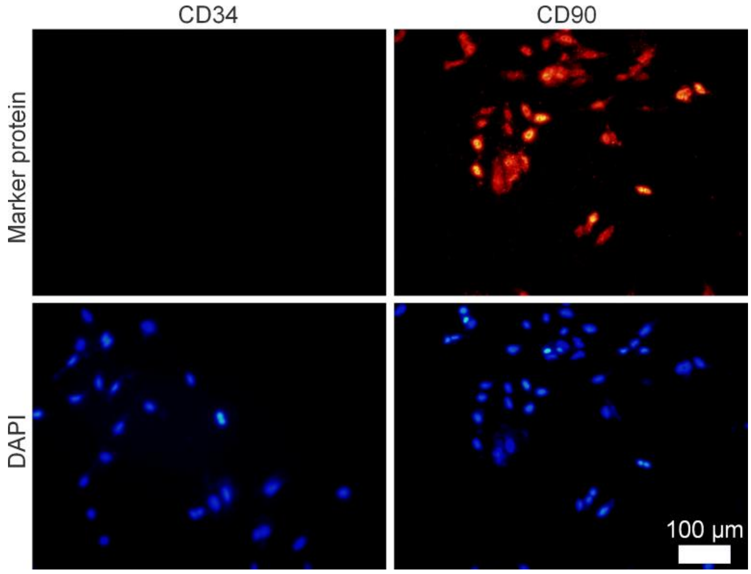

Figure S4. Immunofluorescence staining of stem cell marker proteins CD34 and CD90.

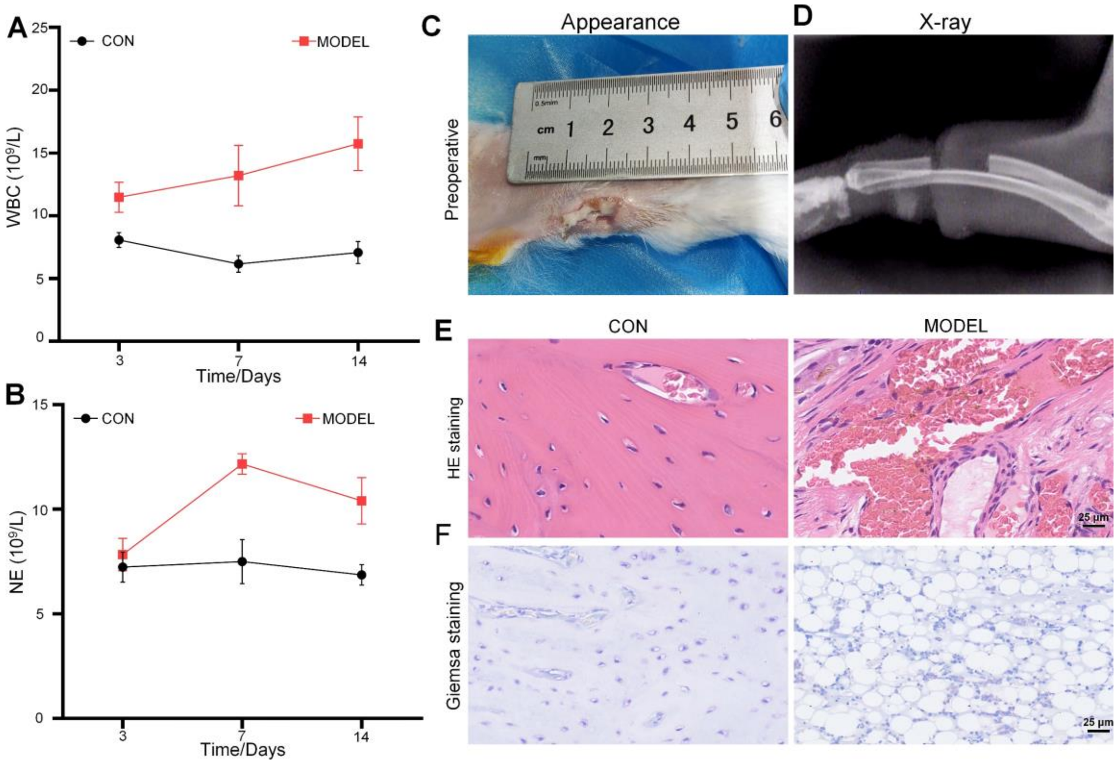

Figure S5. Evaluation of the animal model of infectious bone defect: A-B. Changes in white blood cell (A) and neutrophil (B) content after the preparation of the infection model; C. Local appearance at 2 weeks after surgery; D. X-ray at 2 weeks after surgery; E-F. HE and Giemsa staining at 2 weeks after surgery.
